# Detection of DNA oligonucleotides with base mutations by terahertz spectroscopy and microstructures

**DOI:** 10.1371/journal.pone.0191515

**Published:** 2018-01-24

**Authors:** Mingjie Tang, Mingkun Zhang, Shihan Yan, Liangping Xia, Zhongbo Yang, Chunlei Du, Hong-Liang Cui, Dongshan Wei

**Affiliations:** 1 Chongqing Key laboratory of Multi-Scale manufacturing Technology, Chongqing Institute of Green and Intelligent Technology, Chinese Academy of Sciences, Chongqing, China; 2 College of Instrumentation Science and Electrical Engineering, Jilin University, Changchun, Jilin, China; Oregon State University, UNITED STATES

## Abstract

DNA oligonucleotides with a 5-base mutation at the 3'-terminus were investigated by terahertz (THz) spectroscopy in a marker-free manner. The four single-stranded oligonucleotides with 17nt have been detected with specificity on a microfluidic chip, and corroborated by spectral measurements with split-ring resonators. The number of hydrogen bonds formed between the oligonucleotide and its surrounding water molecules, deemed a key contribution to the THz absorption of biological solutions, was explored by molecular dynamics simulations to explain the experimental findings. Our work underlies the feasibility of THz spectroscopy combined with microstructures for marker-free detection of DNA, which may form the basis of a prospective diagnostic tool for studying genic mutation.

## Introduction

Gene mutation can change the whole structure of a gene through the addition, deletion, or alteration of one or more bases in the DNA sequence. It is generally harmful and will give rise to genetic diseases and cancers, yet it is beneficial for the biological evolution on the other hand. Therefore, detection of the gene mutation is very important for many biomedical applications, such as genetic analysis [1,2] and cancer research [3,4]. To diagnose the gene mutation, it is crucial to detect DNA oligonucleotides generated after mutation. Current techniques for DNA detection, e.g., hybridization assay, usually require complicated and costly fluorescent labeling processes which affect the precision of gene detection due to the degradable labels [5–7]. In recent years, marker-free gene detection techniques are garnering more and more attention and beginning to be recognized as the development trend of gene detection [8,9].

THz spectroscopy has been widely recognized as an extremely attractive technique to detect biomolecules without the requirement of markers, as many vibration, rotation and translation related excitations of biomolecules and weak intermolecular interactions such as hydrogen bonds and van der Waals attraction involve optical transitions in the THz frequency range (0.1–10.0 THz), providing information that is not generally present in IR, NMR and X-ray spectroscopy. A few studies have shown that intense THz pulses could induce cellular and molecular responses [10,11], but THz radiation is almost non-invasive to biological systems in general [12–14]. Therefore, THz spectroscopy is capable of detecting biomolecules according to their structural and conformational changes which are difficult to be captured using other techniques [15].

However, a bottleneck for THz spectroscopy detection of biomolecules has existed since the technique’s inception, as the strong absorption of water in biological solutions may cover up most of the THz signatures of dissolved biomolecules. To overcome this difficulty, some studies [16,17] have found that dispersing solutions in slits of a microchannel can reduce the water content in transmission detection, so as to reduce the background absorption of water effectively. Moreover, if the slit size is further reduced to a scale comparable to the size of the oligonucleotide molecule, the role of the channel wall will enhance the oscillation intensities of some THz characteristic vibration modes [18].

Metamaterials with micro- or nano-gap structures are endowed with strongly localized and enhanced electric field effects, enabling sensitive detection of trace biological and chemical substances [19–28]. Split-ring resonators (SRRs), one such type of metamaterial, composed of periodically arranged, sub-wavelength metallic and/or dielectric elements, are designed to show distinctive electromagnetic properties such as negative refraction [29,30], sub-diffraction limited focusing [31–33], and cloaking [34,35].

In this work, THz time-domain spectroscopy (THz-TDS) measurements are first performed to discriminate the single-stranded 17nt oligonucleotides with 5-base mutations at the 3'-terminus in aqueous solution, based on a microfluidic chip. Then the THz-TDS combined with the split-ring resonators (SRRs) are also employed to discriminate and validate the corresponding dry samples of DNA oligonucleotides at room temperature. Finally, molecular dynamics simulations of four DNA oligonucleotide solutions are performed to elucidate the mechanism responsible for the measured THz spectra.

## Experimental methods

### Experimental preparation

#### Design and preparation of oligonucleotides

The four oligonucleotides with 17nt target 5-base mutations at the 3'-terminus (Table 1) were synthesized and then purified by HPLC (high-performance liquid chromatography) at GenScript Co., Ltd. (Nanjing, China). They were dissolved separately in a TE buffer at pH 7.2 with a concentration of 5 μg/μL.

**Table 1:** Sequences of oligonucleotides investigated in this study.

Oligo ID	Sequences (5'>3')^a^
Ter-5A	TTAGGGTTAGGGAAAAA
Ter-5C	TTAGGGTTAGGGCCCCC
Ter-5T	TTAGGGTTAGGGTTTTT
Ter-5G	TTAGGGTTAGGGGGGGG

^a^The underlined letters indicate different bases.

#### Design and preparation of microstructures

**Fig 1:**
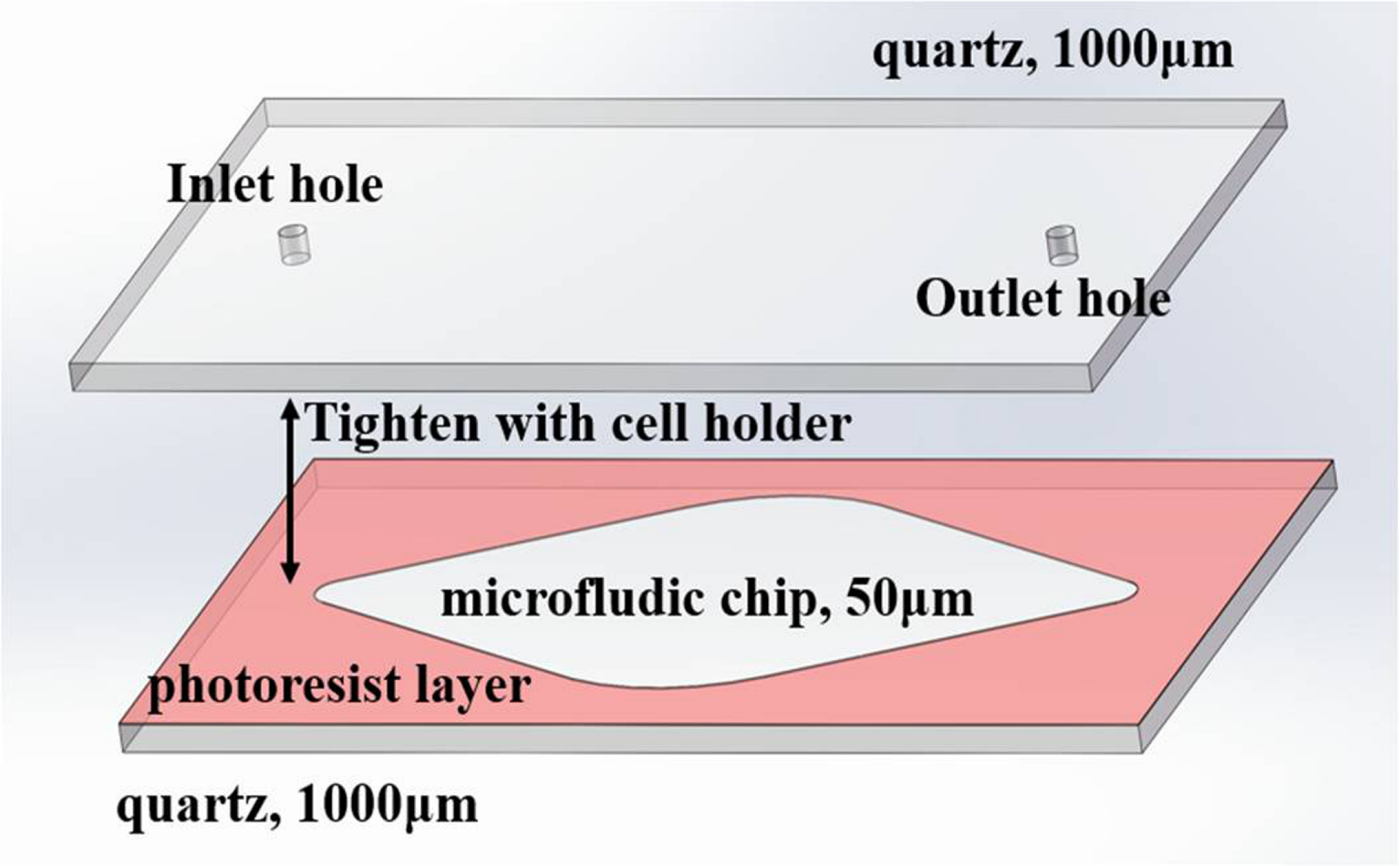
A schematic presentation of the microfluidic chip. The chip involves a 17-mm-long filleted rhombus with a depth of 50 μm on one of the two quartz plates was fabricated by lithography. The inlet/outlet circular holes with a diameter of 1 mm were fabricated by laser drilling.

A microfluidic chip based on SU-8 photoresist was designed and fabricated as a liquid sample cell for THz measurements of the buffer and these four DNA oligonucleotide solutions. The chip involves 2 pieces of 1-mm-thick quartz windows. By spin-coating a 50-μm-thick SU-8 2050 photoresist layer on one of the two quartz plates, a 17-mm-long filleted rhombus with a depth of 50 μm on the quartz plate was fabricated by lithography. Then by marking the inlet/outlet hole positions close to the left and right ends of the filleted rhombus, two circular holes with a diameter of 1 mm were fabricated by laser drilling. Subsequently, by covering the photoresist layer with the other piece of quartz, the detachable microfluidic chip was prepared as shown in Fig 1. During the using of the microfluidic chip, a standard Bruker liquid cell holder was used to tighten two quartz plates for preventing the leakage of the liquid sample. Before each THz spectral measurement, the liquid sample was gently injected into the rhombic reservoir through the inlet hole, and fully immersed into the reservoir by the syringe in order to ensure the quantity of the injected liquid is equal to the volume of the reservoir around 12.0 μL.

**Fig 2:**
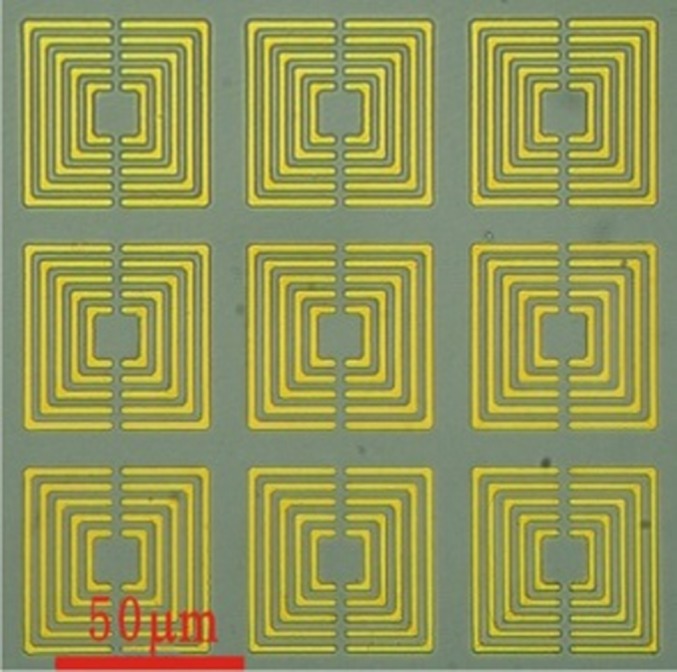
An optical microscope image of the SRRs. There are 20 nm thick Cr (Chromium) and 200 nm thick Au (gold) metal films on the Si substrate. The SRRs have a period of 60 μm. The gap size between two adjacent open square rings is equivalently 2.5 μm. The linewidth of the open square ring is 2 μm and the line length for the maximum open square ring is 50 μm.

The SRRs composed of metallic square ring arrays, each of which was formed by five open square rings with different sizes, were fabricated by a traditional photo-lithography technique on a high resistance silicon substrate with a thickness of 400 μm, the same with those used in our previous bovine serum albumin protein detection experiments [36]. In order to pattern the SRRs structure, 20 nm thick Cr (Chromium) and 200 nm thick Au (gold) metal films were successively deposited on the Si substrate. The SRRs have a period of 60 μm. The gap size between two adjacent open square rings is equivalently 2.5 μm. The linewidth of the open square ring is 2 μm and the line length for the maximum open square ring is 50 μm, as depicted in Fig 2.

#### THz spectroscopy measurement

A commercial THz-TDS system (Advanced Photonix, Inc., T-Ray 5000, USA) was utilized to measure the THz spectra of the DNA oligonucleotide solution in the microfluidic chip and to monitor the THz resonance peak shifts of dry oligonucleotide samples covering on the SRRs metamaterial. The femtosecond pulse was produced by a Ti-sapphire laser with a repetition rate of 100 MHz, a central wavelength of 1064 nm, and a duration of <100 fs. This pulse was divided into two parts by a polarizing beam splitter, one as the probe beam directly irradiating on the photoconductive antenna (PCA), the other as the pump light gathering on the other PCA that had been biased electrically to generate THz radiation with an average power of 130 nW, focusing on and transmitting through the microstructure. The THz signal irradiated on the second PCA was sampled discretely by the probe light to acquire the time-domain waveforms, subsequently fast-Fourier transformed to frequency domain. A relative humidity of 3% was maintained by the purge of nitrogen gas during measurements.

#### Molecular dynamics simulation

A molecular dynamics simulation package AMBER 12 [37] was used to simulate these DNA oligonucleotide chains at 293 K. Four different linear oligonucleotide chains whose sequences are shown in Table 1 with their corresponding counter-ions Na^+^ were built using ff12SB force field [38]. Each of them was energy-minimized and heated to 293 K in vacuum, then the system was maintained around the desired temperature for 10 ns to obtain a stable configuration. Subsequently, each oligonucleotide chain was solvated in explicit TIP3P water model in a cubic box with periodic boundary conditions. After that, an energy minimization was conducted using a steepest descent algorithm followed by a conjugate gradient algorithm. Then an NPT ensemble simulation at 1.0 bar and 293 K was performed to obtain the DNA solution system with an approximate density of 0.998 g/cm^-3^. Lastly, an NVT ensemble simulation for 5 ns and an NVE ensemble simulation for 20 ns were successively conducted to make the system reach a final stable state for the production analysis. The time step in all the simulations was 1.0 fs.

## Results and discussion

### THz spectroscopy analysis of oligonucleotides in aqueous solution based on the microfluidic chip

THz spectra of the four oligonucleotides in TE buffer solution loaded into the microfluidic chip were measured using the THz-TDS system with a frequency resolution of 12.5 GHz. Each oligonucleotide solution sample was repeatedly measured three times on different days to minimize the effect of fluctuation in instrument performance. Before each measurement, the microfluidic chip was cleaned by alcohol and deionized water and dried in nitrogen.

**Fig 3:**
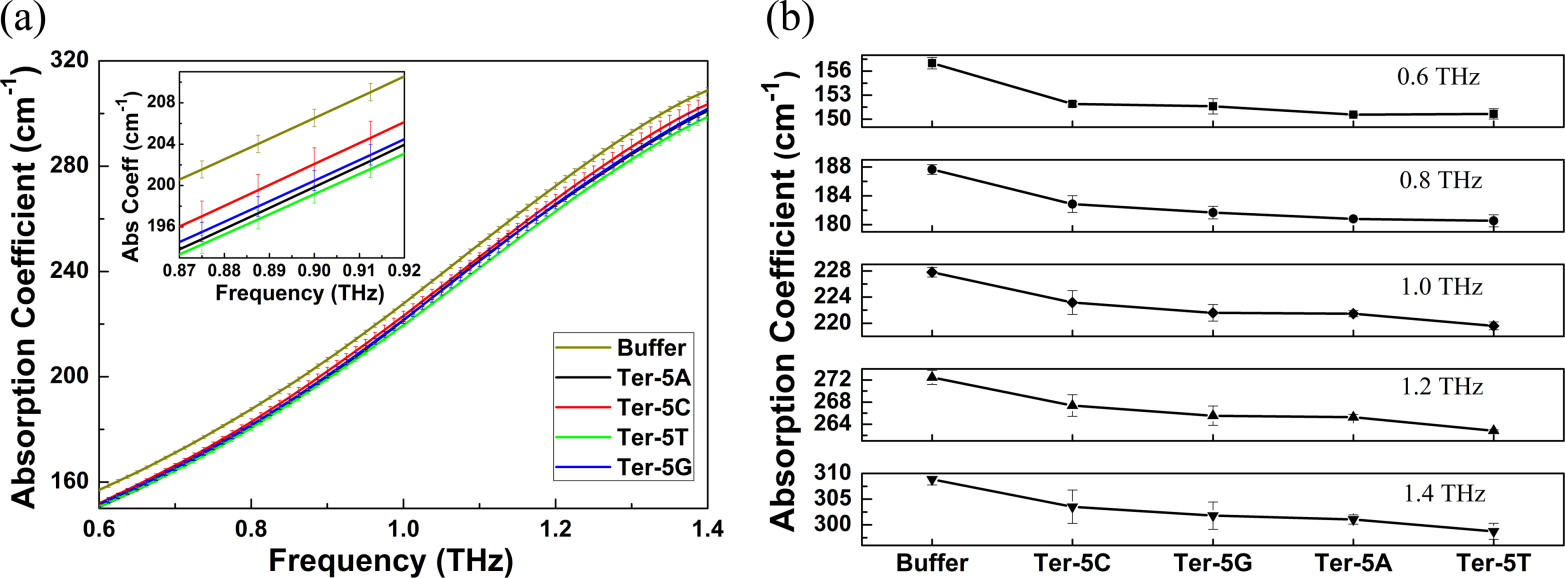
(a) Absorption coefficients of TE buffer and these four oligonucleotide solutions at 0.6–1.4 THz. The inset shows the amplified spectra at a narrow frequency band of 0.87–0.92 THz to highlight the error bars and the frequency resolution clearly; (b) Absorption coefficients of these samples at 0.6, 0.8, 1.0, 1.2 and 1.4THz, respectively.

The absorption coefficients of TE buffer and each sample in the frequency range of 0.6–1.4 THz are shown in Fig 3A. From this figure, we can see that there is no characteristic absorption peak for all samples and the THz absorption coefficient of the buffer is prominently higher than those of the four oligonucleotide solutions, which is in agreement with the two-components excluded volume model [39,40] since the THz absorption of the bulk water is higher than those of the DNA oligonucleotide molecules. While the difference in the THz absorption between the four oligonucleotide solutions is not notable. To better describe the difference, the inset in Fig 3A shows the amplified absorption spectra of TE buffer and these four oligonucleotides at 0.87–0.92 THz. At such a narrow frequency band, we can clearly see that the absorption coefficients of the four oligonucleotide solutions are in such an order: Ter-5T < Ter-5A < Ter-5G < Ter-5C. Not only at this frequency band, the absorption coefficients of these oligonucleotides at 0.6, 0.8, 1.0, 1.2 and 1.4 THz as shown in Fig 3B are also in the same order with that at 0.87–0.92 THz. Although there is some overlapping of the error bars from Fig 3A, the four oligonucleotides can nevertheless be differentiated from the averaged absorption intensity over all frequencies, given that all of these oligonucleotide solutions are measured under the same experimental conditions.

It is worth noting that the difference in absorption coefficient between four oligonucleotide solutions is not prominent due to the strong water absorption. To verify the difference is meaningful in statistics and can be used to discriminate the four samples, the one-way analysis of variance (ANOVA) is performed since the ANOVA test can be well used to compare means of two or more samples using the F distribution [41]. To perform the test, the three-time repeated measured absorption coefficient data around each frequency *f* (*f* ± 0.1 THz) of these four oligonucleotide solutions are used. The statistic *F* value from the one-way ANOVA test is calculated as below [41],

$$F=\frac{\overset{m}{\underset{j=1}{\sum }}n{\left(\left\langle Z_{j}\rangle -\langle Z\right\rangle \right)}^{2}/(m-1)}{\overset{m}{\underset{j=1}{\sum }}\overset{n}{\underset{i=1}{\sum }}{\left(Z_{ij}-\langle Z_{j}\rangle \right)}^{2}/(nm-m)},$$ (1)

where *m* is the number of these oligonucleotide solutions with *m* = 4, *n* is the number of absorption coefficient data for one of the oligonucleotide solutions around each frequency, *Z*_*ij*_ is the absorption coefficient of *i*-th data point for *j*-th oligonucleotide solution, <*Z*_*j*_> is the mean of the absorption coefficient for *j*-th oligonucleotide solution and <Z> is the overall mean of all absorption coefficient data of these four oligonucleotide solutions. The calculated *F* values around 0.6, 0.8, 1.0, 1.2, 1.4 THz are given in Table 2. From the table, we can see that all of these *F* values are larger than 1.0, indicating the difference in the means of the absorption coefficients between the four oligonucleotide solutions is significant and meaningful in statistics.

**Table 2:** F values from the ANOVA analysis at different frequencies.

Frequency	0.6 THz	0.8 THz	1.0 THz	1.2 THz	1.4 THz
F value	2.6	3.1	3.1	3.9	1.5

**Fig 4:**
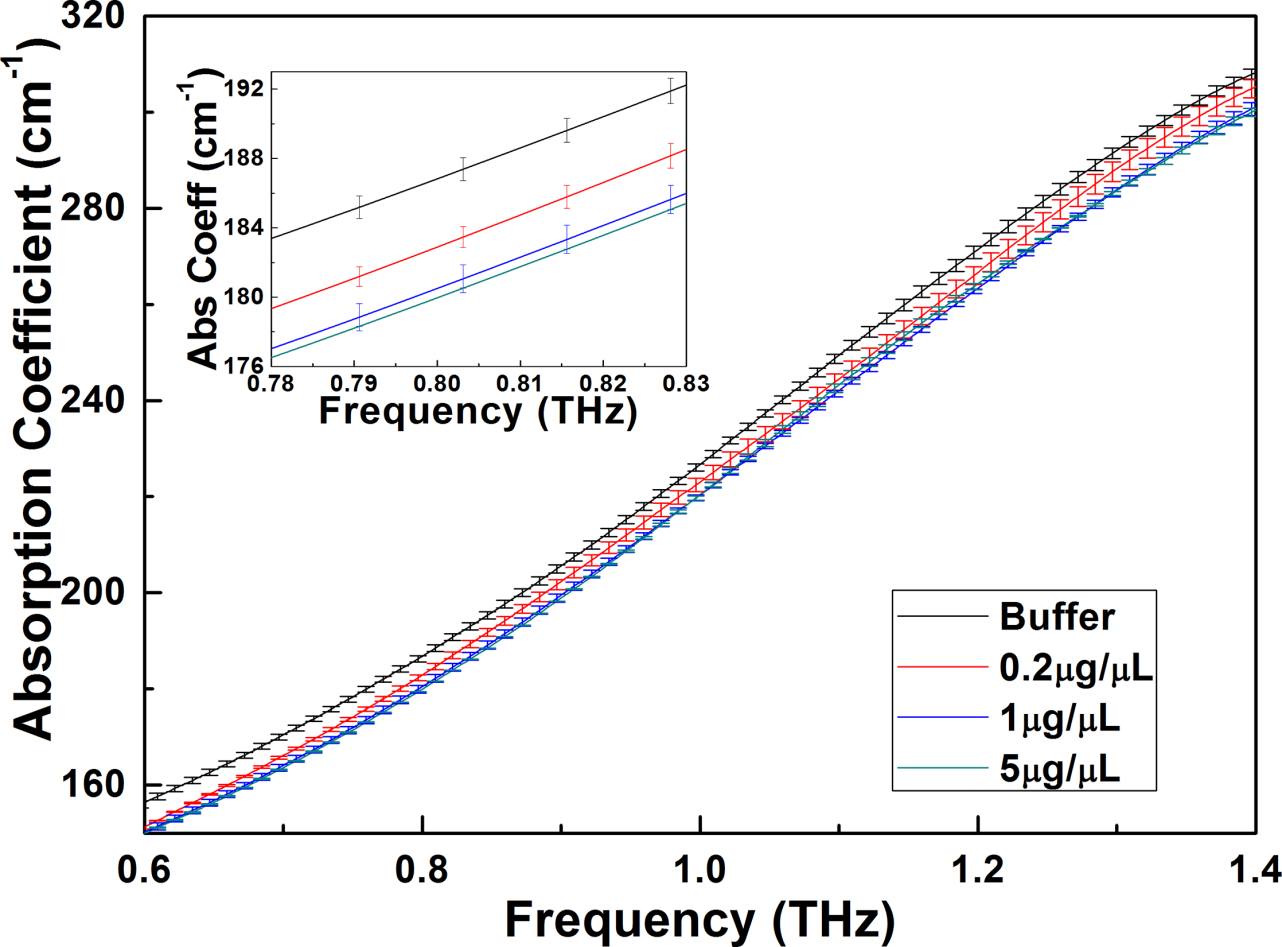
Absorption coefficients of TE buffer and the concentration dependence of the Ter-5A at 0.6–1.4 THz. The inset shows the amplified spectra at a narrow frequency band of 0.78–0.83 THz to highlight the error bars and the frequency resolution clearly.

In addition, the concentration effect on the THz absorption of the DNA oligonucleotide solutions is also considered. THz absorption spectra of Ter-5A sample at two other concentrations of 1.0 and 0.2 μg/μL are also measured. The comparison between the Ter-5A sample at these three concentrations and the TE buffer is plotted and shown in Fig 4. From this figure, the variation trends of absorption coefficient vs frequency for the Ter-5A solution at 1.0 and 0.2 μg/μL are the same with that at 5.0 μg/μL except that the magnitude of the absorption coefficient changes. At the same frequency, the absorption coefficient decreases with the increase of the concentration, which is in agreement with most reported results for biomolecular solutions [42,43]. Furthermore, we also measure THz spectral measurements of all four DNA oligonucleotide solutions at the same concentration of 1.0 μg/μL and find the absorption coefficient at 1.0 μg/μL has the same variation trend with that at 5.0 μg/μL. Therefore, the concentration will not affect our detection results.

#### THz spectroscopy analysis of dried oligonucleotide samples in air based on SRRs

To investigate the resonance peak shift after the oligonucleotide sample deposited on the SRRs metamaterial, and in order to guarantee the equal film thickness for each oligonucleotide measurement, we elaborately prepared the measurements. Each oligonucleotide solution with the same volume of 20.0 μL was firstly dripped and coated evenly on the clean and optical horizontal surface of the SRRs. Before the sample solution was dripped, the SRRs chip was placed into a clamped groove to prevent the overflow of the sample solution. Then each nucleotide solution was dried for 5 h in nitrogen gas at room temperature prior to the THz spectroscopy measurement.

**Fig 5:**
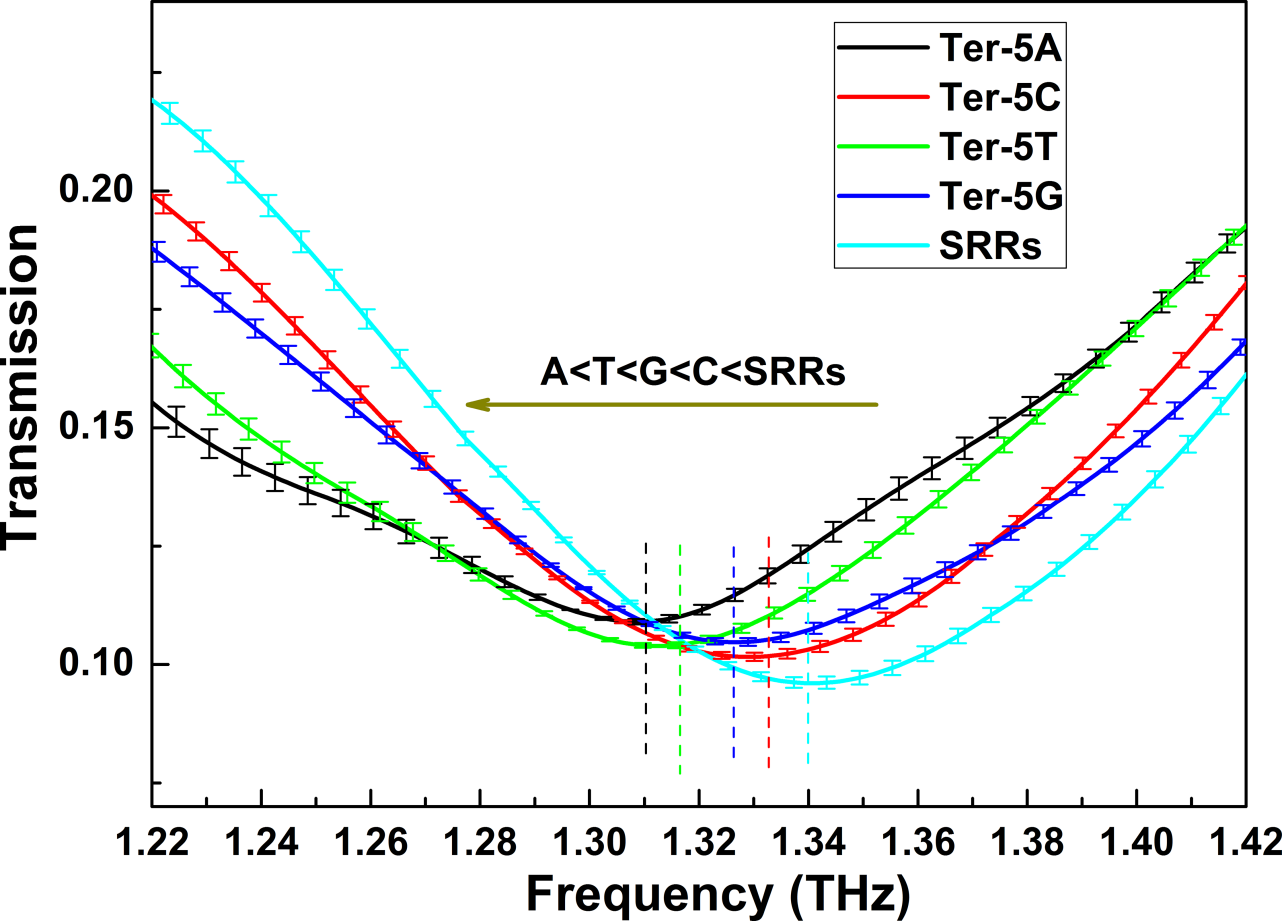
THz transmission spectra of the SRRs and the dry oligonucleotides on SRRs. The resonant frequency peak for each spectrum is marked with a vertical dash.

The mean transmission intensities of the four dry oligonucleotide samples on the SRRs from three independent measurements were shown in Fig 5. Before each measurement, the SRRs chip was also cleaned by alcohol and deionized water and dried in nitrogen gas. The resonance peak of the blank SRRs chip was located at 1.340 THz. However, when the SRRs chip is covered with the oligonucleotide, the resonant peak position is red-shifted compared with that of the blank SRRs. The SRRs can be modeled as an LC oscillator with a resonant frequency given byω = 1/sqrt(LC), where *L* is the equivalent inductance, and *C* is the equivalent capacitance of the SRRs [36]. The red-shift can be interpreted by the change of capacitance in the gap area, which is proportional to the effective dielectric constant of the detected aggregate of oligonucleotide molecules, given the dripped quantity and the thickness of the four oligonucleotide samples are the same. Apparently, the oligonucleotides attached to the SRRs caused the effective dielectric constant to increase, resulting in the red-shift of the resonant frequency.

The resonant frequencies of the oligonucleotides (Ter-5A, Ter-5C, Ter-5T, Ter-5G) were determined to be 1.310, 1.333, 1.317 and 1.326 THz, respectively, with a standard deviation of 0.1% after averaging over three independent measurements for each sample. Due to the 12.5 GHz resolution of the used THz-TDS system, to precisely determine the resonant frequencies of these oligonucleotides, an interpolation method is used to increase the frequency points around the resonant frequency for each sample. As we can see, the resonant frequencies of Ter-5C and Ter-5G were higher than those of the Ter-5A and Ter-5T and differences between these resonance frequencies are discernible. The surface plasmon resonance (SPR) theory predicts a close relation between the resonant frequency and the effective dielectric constant, based on which we can conclude that the four oligonucleotides have different dielectric constants and the dielectric constants of Ter-5A and Ter-5T are approximately equal. It is worth noting that the approximate red-shifts of resonant frequencies for Ter-5A and Ter-5T oligonucleotides observed here are also consistent with the approaching THz absorption coefficients for the Ter-5A and Ter-5T oligonucleotides at lower frequencies (< 0.8 THz) as shown in Fig 3A and 3B.

#### MD simulation of four oligonucleotide solutions

Since THz spectroscopy mainly manifests weak molecular interactions including primarily hydrogen bonds and van der Waals force, the number and intensity of hydrogen bonds are important factors influencing THz absorption intensity [44,45]. To understand the above detection results by the THz spectroscopies and the microstructures, MD simulations were performed to study the hydrogen bond behavior of these four oligonucleotide chains in aqueous solution.

Taking the last 10 ns trajectory from the NVE ensemble simulation discussed earlier in Section 2.3, we first calculated the hydrogen bond number in the simulation box. Since the sizes of the four DNA molecules and the simulation box for each oligonucleotide are almost the same, the intra-molecular hydrogen bonds from the DNA molecule and the hydrogen bonds formed between water molecules in the simulation box are almost the same for each sample. Moreover, difference in absorption coefficients of biomolecular solutions is mainly related to the hydrogen bond network formed by the biomolecule and its surrounding water [46,47]. Therefore, only hydrogen bonds formed between the oligonucleotide and its surrounding water molecules are counted here. The hydrogen bond is defined with a maximum donor-acceptor distance of 3.5 Å and a minimum donor-H-acceptor angle of 135°, which is a regular definition in Amber software.

**Fig 6:**
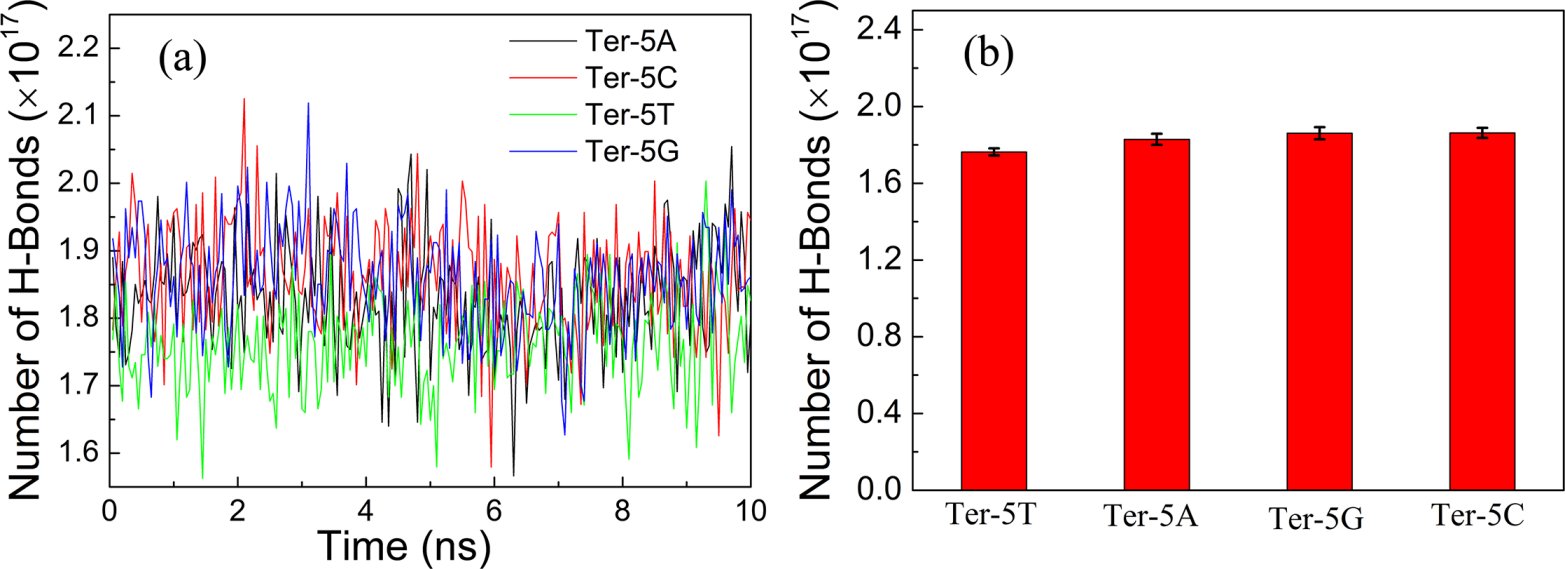
(a) The normalized number of hydrogen bonds formed between the oligonucleotide and surrounding water molecules at different MD simulation times. (b) The average normalized number of hydrogen bonds for these four oligonucleotide solutions.

After calculating the hydrogen bond number in the simulation box for each oligonucleotide solution, the hydrogen bond number was normalized to a magnitude which corresponds to the same mass concentration in experiment with a volume of 1 uL according to the following relation:

$${\tilde{N}}_{HB}=N_{HB\_MD}\times \frac{cN_{A}}{M},$$ (2)

where *N*_*HB_MD*_ is the number of hydrogen bonds in the simulation box, *c* is the mass concentration in experiment and *M* is the molar mass of the DNA nucleotide. The statistical results of the number of hydrogen bonds for each oligonucleotide in solution at different simulation time are depicted in Fig 6A and the average results of hydrogen bonds over the last 10 ns NVE simulations are shown in Fig 6B. From Fig 6B, it can be seen that the number of hydrogen bonds in the four solution systems are in such an order: Ter-5T < Ter-5A < Ter-5G < Ter-5C. More hydrogen bonds between the biomolecule and its surrounding water implies more hydration water, which may give rise to stronger THz absorption [39,40,48]. This is in good agreement with our above THz measurements illustrated in Fig 3 and previous experiment results [45].

## Conclusions

In this study, measurement techniques combining the THz spectroscopy and microstructures were proposed to try to detect the DNA oligonucleotides with 5-base mutation. Based on the experimental results and the simulation analysis, we can draw the following conclusions: (1) A microfluidic chip can be used as a carrier device suitable for THz detection of biomolecular solutions to reduce the strong absorption of water in the THz frequency range; (2) The four oligonucleotides with the 5-base mutation (Ter-5A, Ter-5C, Ter-5T, Ter-5G) can be detected with specificity according to their different THz absorption spectra. (3) The four oligonucleotides were found to exhibit different red-shifts in resonance frequency because of their different effective dielectric constants; (4) Difference in the number of hydrogen bonds formed by the oligonucleotide molecules and their surrounding water molecules can be well used to interpret the difference in the THz absorption coefficients of these four oligonucleotide solutions. Therefore, the investigation results reported in this work indicate that THz spectroscopy may be an effective and marker-free technique to explore and diagnose the genic mutation of DNA molecules.

## Supporting information

S1 File Absorption coefficient datasheet of TE buffer and four oligonucleotide solutions at 0.6–1.4 THz. (PDF)

S2 File Absorption coefficient datasheet of TE buffer and four oligonucleotide solutions at 0.6, 0.8, 1.0, 1.2 and 1.4THz. (PDF)

S3 File Absorption coefficient datasheet of TE buffer and Ter-5A at three different concentrations at 0.6–1.4 THz. (PDF)

S4 File THz transmission spectral datasheet of the SRRs and the dry oligonucleotides on SRRs. (PDF)

S5 File Hydrogen bond datasheet at different simulation times. (PDF)

S6 File Average hydrogen bond datasheet for four sample solutions. (PDF)
