# Supplementary material for: Detection of DNA oligonucleotides with base mutations by terahertz spectroscopy and microstructures
Source: PLoS One. 2018 Jan 24;13(1):e0191515. doi: 10.1371/journal.pone.0191515 (PMC5783420; doi:10.1371/journal.pone.0191515)
Supplement: S1 File — (PDF) [file pone.0191515.s001.pdf]

Supporting Information

Plotting data of Fig. 3a

Frequency (THz)	Absorption Coefficient(cm^{-1})									
	Buffer	SD	Ter-5A	SD	Ter-5C	SD	Ter-5T	SD	Ter-5G	SD
0.6	156.9963	0.70669	150.5643	0.08453	151.907	0.3015	150.6561	0.64726	151.6095	0.95336
0.6125	158.649	0.6778	152.1518	0.09003	153.6454	0.35403	152.2486	0.67506	153.1944	0.94573
0.625	160.3367	0.65135	153.7779	0.09295	155.4	0.41111	153.8739	0.70313	154.8181	0.93383
0.6375	162.0189	0.62793	155.4031	0.09395	157.131	0.47093	155.4916	0.73013	156.4406	0.91924
0.65	163.739	0.60788	157.0723	0.09439	158.8825	0.53036	157.1456	0.75369	158.1058	0.90421
0.6625	165.5249	0.59155	158.8141	0.09477	160.6846	0.58794	158.8648	0.77297	159.842	0.89016
0.675	167.368	0.57928	160.6196	0.09473	162.5316	0.64392	160.6405	0.78824	161.6397	0.87816
0.6875	169.263	0.57124	162.4828	0.09326	164.4219	0.69903	162.4697	0.8007	163.4937	0.8678
0.7	171.1639	0.5675	164.3571	0.08983	166.3128	0.75358	164.3086	0.81092	165.3579	0.85901
0.7125	173.0718	0.56791	166.2427	0.08416	168.2082	0.80778	166.1589	0.81955	167.2326	0.85215
0.725	175.0413	0.57228	168.1944	0.07607	170.1655	0.86095	168.0769	0.82703	169.1725	0.84704
0.7375	177.0706	0.58055	170.2105	0.06596	172.1852	0.91271	170.061	0.83324	171.1754	0.84375
0.75	179.1454	0.59281	172.2771	0.05424	174.2565	0.96371	172.0978	0.83828	173.2264	0.84301
0.7625	181.2423	0.60857	174.3708	0.04178	176.3591	1.01426	174.1649	0.84209	175.303	0.84534
0.775	183.3322	0.62751	176.4619	0.02955	178.4645	1.06428	176.232	0.8438	177.3756	0.8511
0.7875	185.4587	0.6495	178.5935	0.01787	180.6162	1.1136	178.3412	0.84366	179.487	0.86024
0.8	187.6561	0.67409	180.7996	0.0072	182.8478	1.16305	180.5254	0.84406	181.6707	0.87167
0.8125	189.9017	0.70026	183.0567	0.00257	185.1347	1.21336	182.7595	0.8465	183.9031	0.88423
0.825	192.1785	0.72703	185.348	0.00938	187.4594	1.26434	185.0253	0.85105	186.1682	0.89571
0.8375	194.4594	0.75353	187.6468	0.01489	189.7927	1.31503	187.2929	0.85634	188.4393	0.90594
0.85	196.7524	0.77865	189.9615	0.01825	192.1398	1.36596	189.5677	0.86203	190.7242	0.91552
0.8625	199.115	0.80167	192.3483	0.01811	194.5543	1.41759	191.9041	0.86748	193.0794	0.92456
0.875	201.5483	0.8211	194.809	0.01096	197.0344	1.46863	194.3012	0.87003	195.505	0.93464
0.8875	204.0232	0.83564	197.3156	0.00635	199.5494	1.51818	196.729	0.86863	197.9722	0.94696
0.9	206.5197	0.84393	199.8481	0.0308	202.0777	1.56582	199.167	0.86323	200.4612	0.96134
0.9125	209.0247	0.84584	202.3917	0.0648	204.6022	1.61198	201.6006	0.85376	202.9568	0.97781
0.925	211.5774	0.8414	204.9833	0.10496	207.1604	1.65781	204.069	0.84106	205.4966	0.99649
0.9375	214.2118	0.83055	207.6573	0.15143	209.7867	1.70194	206.6076	0.82462	208.115	1.01839

0.95	216.8952	0.81461	210.3825	0.20434	212.4493	1.74127	209.1846	0.80096	210.7808	1.04669
0.9625	219.596	0.79539	213.1277	0.26134	215.1188	1.77456	211.7709	0.7676	213.464	1.08305
0.975	222.2985	0.7747	215.8762	0.32123	217.7804	1.79996	214.3537	0.72374	216.1475	1.1312
0.9875	225.017	0.75476	218.6408	0.38585	220.4488	1.81545	216.949	0.66995	218.8429	1.19469
1	227.799	0.73907	221.4668	0.45755	223.174	1.81799	219.6049	0.60601	221.5971	1.27391
1.0125	230.6368	0.73094	224.349	0.53609	225.9553	1.8049	222.3166	0.53071	224.4049	1.36771
1.025	233.4792	0.73458	227.2357	0.61615	228.7454	1.78065	225.0365	0.44861	227.2169	1.47141
1.0375	236.3011	0.75227	230.0959	0.69122	231.5198	1.75195	227.741	0.36619	230.0079	1.57936
1.05	239.1044	0.78396	232.9273	0.75812	234.2815	1.72018	230.4325	0.28574	232.7792	1.68934
1.0625	241.9224	0.82714	235.762	0.81604	237.0662	1.68496	233.1452	0.20788	235.5659	1.79883
1.075	244.7807	0.87841	238.626	0.86561	239.9002	1.64515	235.9046	0.13231	238.3952	1.90453
1.0875	247.6408	0.93451	241.4785	0.90354	242.7432	1.60441	238.6712	0.06122	241.2286	2.00248
1.1	250.4647	0.99096	244.2753	0.92585	245.5552	1.57264	241.4087	0.01363	244.0257	2.08314
1.1125	253.256	1.04647	247.0145	0.93385	248.3362	1.55261	244.1187	0.04645	246.7844	2.14149
1.125	256.0364	1.10102	249.7177	0.93045	251.1057	1.5456	246.8186	0.07595	249.5233	2.17411
1.1375	258.8345	1.15325	252.4146	0.91315	253.8929	1.55572	249.5374	0.08674	252.2726	2.17493
1.15	261.6358	1.19867	255.095	0.87667	256.6837	1.58845	252.2659	0.07576	255.0206	2.14237
1.1625	264.3934	1.23443	257.7122	0.81793	259.4247	1.65105	254.9573	0.03899	257.7179	2.07619
1.175	267.1011	1.25956	260.2588	0.73764	262.1051	1.73998	257.5999	0.02424	260.3565	1.98165
1.1875	269.7836	1.27455	262.7658	0.64064	264.7506	1.84132	260.2151	0.09895	262.9592	1.86911
1.2	272.4692	1.27941	265.2664	0.53078	267.3872	1.94887	262.8282	0.18469	265.5519	1.74604
1.2125	275.1697	1.27411	267.778	0.40919	270.0268	2.06328	265.4505	0.27877	268.1453	1.61739
1.225	277.8456	1.26145	270.2647	0.27736	272.6333	2.18601	268.0444	0.38356	270.7014	1.48972
1.2375	280.4599	1.24333	272.6919	0.1415	275.1756	2.31515	270.5769	0.49809	273.1852	1.36832
1.25	283.0234	1.22302	275.0778	0.03245	277.6692	2.44032	273.0569	0.61864	275.6117	1.26484
1.2625	285.5511	1.20061	277.4493	0.11814	280.1341	2.55831	275.5003	0.74201	278.0072	1.18887
1.275	288.0522	1.1743	279.8242	0.24161	282.587	2.67123	277.9249	0.86393	280.3962	1.14539
1.2875	290.5152	1.14211	282.1932	0.36032	285.0178	2.77946	280.3211	0.9815	282.7742	1.14146
1.3	292.9024	1.10948	284.5157	0.46804	287.3854	2.87878	282.6442	1.09011	285.1004	1.17852
1.3125	295.2074	1.08226	286.7835	0.56161	289.6825	2.95858	284.8837	1.18531	287.3678	1.25829
1.325	297.4448	1.06092	289.0152	0.64032	291.9249	3.01392	287.0645	1.26501	289.5947	1.38319
1.3375	299.6156	1.04476	291.2107	0.70779	294.1101	3.0587	289.1974	1.33564	291.7851	1.54656
1.35	301.7079	1.03343	293.3591	0.76658	296.2266	3.1084	291.2784	1.40552	293.936	1.73885
1.3625	303.6842	1.02867	295.4233	0.81492	298.2371	3.16248	293.278	1.47162	296.0184	1.95301
1.375	305.5181	1.03042	297.3759	0.85223	300.1116	3.21083	295.1735	1.52231	298.0083	2.1827
1.3875	307.232	1.04418	299.2414	0.87823	301.8684	3.24159	296.9862	1.55142	299.9297	2.42135

1.4	308.8443	1.07896	301.0369	0.89268	303.5204	3.25326	298.7356	1.56065	301.7965	2.65743
