# Supplementary material for: Detection of DNA oligonucleotides with base mutations by terahertz spectroscopy and microstructures
Source: PLoS One. 2018 Jan 24;13(1):e0191515. doi: 10.1371/journal.pone.0191515 (PMC5783420; doi:10.1371/journal.pone.0191515)
Supplement: S2 File — (PDF) [file pone.0191515.s002.pdf]

Plotting Data of Fig. 3b

	0.6 THz	SD	0.8 THz	SD	1.0 THz	SD	1.2 THz	SD	1.4 THz	SD
Buffer	156.9963	0.70669	187.6561	0.67409	227.799	0.73907	272.4692	1.27941	308.8443	1.07896
Ter-5C	151.907	0.3015	182.8478	1.16305	223.174	1.81799	267.3872	1.94887	303.5204	3.25326
Ter-5G	151.6095	0.95336	181.6707	0.87167	221.5971	1.27391	265.5519	1.74604	301.7965	2.65743
Ter-5A	150.5643	0.08453	180.7996	0.0072	221.4668	0.45755	265.2664	0.53078	301.0369	0.89268
Ter-5T	150.6561	0.64726	180.5254	0.84406	219.6049	0.60601	262.8282	0.18469	298.7356	1.56065
