# Supplementary material for: Detection of DNA oligonucleotides with base mutations by terahertz spectroscopy and microstructures
Source: PLoS One. 2018 Jan 24;13(1):e0191515. doi: 10.1371/journal.pone.0191515 (PMC5783420; doi:10.1371/journal.pone.0191515)
Supplement: S3 File — (PDF) [file pone.0191515.s003.pdf]

Plotting Data of Fig. 4

Frequency (THz)	Absorption Coefficient(cm^{-1})							
	Buffer	SD	0.2μg/μL	SD	1μg/μL	SD	5μg/μL	SD
0.6	156.3906	0.71792	151.31	0.02232	150.2451	0.72395	149.9835	0.08195
0.60313	156.7919	0.71037	151.7541	0.03371	150.6367	0.7203	150.3681	0.08377
0.60625	157.1969	0.70291	152.2009	0.04486	151.0321	0.71683	150.7564	0.08544
0.60938	157.606	0.69555	152.6507	0.05577	151.4319	0.71353	151.1491	0.08697
0.6125	158.0196	0.68831	153.104	0.06641	151.8363	0.71041	151.5463	0.08836
0.61562	158.4373	0.68121	153.5604	0.0768	152.245	0.70746	151.9479	0.08958
0.61875	158.8582	0.67425	154.0189	0.08694	152.6573	0.70471	152.353	0.09064
0.62187	159.2811	0.66745	154.4784	0.09683	153.0718	0.70216	152.7601	0.09153
0.625	159.7043	0.66083	154.9373	0.10649	153.487	0.69981	153.168	0.09225
0.62813	160.1268	0.65438	155.3943	0.11592	153.9017	0.69765	153.5753	0.09281
0.63125	160.5479	0.64812	155.8487	0.12514	154.3152	0.69569	153.9816	0.09323
0.63438	160.9677	0.64206	156.3006	0.13417	154.7275	0.6939	154.3867	0.09353
0.6375	161.3869	0.6362	156.7504	0.14301	155.1394	0.69229	154.7916	0.09375
0.64063	161.8067	0.63055	157.1995	0.15168	155.5519	0.69083	155.1974	0.0939
0.64375	162.2285	0.62511	157.649	0.16019	155.9666	0.68954	155.6056	0.09403
0.64687	162.6537	0.61987	158.1004	0.16853	156.3847	0.68842	156.0177	0.09414
0.65	163.0832	0.61485	158.5545	0.17671	156.8072	0.68746	156.4344	0.09424
0.65312	163.5174	0.61005	159.0119	0.18473	157.2345	0.68668	156.8563	0.09434
0.65625	163.9562	0.60548	159.4724	0.19262	157.6666	0.68606	157.2833	0.09444
0.65938	164.3993	0.60114	159.9357	0.20036	158.103	0.68561	157.715	0.09454
0.6625	164.8462	0.59705	160.4013	0.208	158.5433	0.68532	158.1509	0.09464
0.66563	165.2965	0.59322	160.869	0.21553	158.9872	0.68517	158.5906	0.09474
0.66875	165.75	0.58963	161.3387	0.22298	159.4343	0.68516	159.0339	0.09482
0.67188	166.2069	0.58631	161.8105	0.23037	159.8849	0.6853	159.481	0.09488
0.675	166.6674	0.58324	162.2849	0.2377	160.3393	0.68558	159.9321	0.0949
0.67812	167.1318	0.58042	162.7623	0.245	160.7978	0.68602	160.3875	0.09485
0.68125	167.6003	0.57787	163.2429	0.25225	161.2606	0.68662	160.8474	0.09471
0.68437	168.0725	0.57559	163.7266	0.25948	161.7274	0.68739	161.3113	0.09447
0.6875	168.5476	0.57357	164.2126	0.2667	162.1975	0.68832	161.7785	0.09411
0.69063	169.0244	0.57183	164.6996	0.27391	162.6696	0.68941	162.2477	0.09363
0.69375	169.5016	0.57037	165.1866	0.28114	163.1424	0.69067	162.7177	0.09302
0.69688	169.9782	0.56917	165.6725	0.28841	163.6148	0.69208	163.1873	0.09228
0.7	170.4535	0.56824	166.1567	0.29575	164.0862	0.69366	163.6559	0.09143
0.70313	170.9274	0.56758	166.6392	0.30316	164.5566	0.69539	164.1235	0.09045
0.70625	171.4006	0.56717	167.1207	0.31067	165.0265	0.69729	164.5906	0.08934
0.70937	171.8742	0.56702	167.6023	0.31828	165.497	0.69934	165.0584	0.08808
0.7125	172.3495	0.56713	168.0854	0.326	165.9694	0.70154	165.5281	0.08667
0.71562	172.8281	0.56748	168.5715	0.33382	166.4453	0.7039	166.0014	0.0851
0.71875	173.3109	0.56809	169.0618	0.34174	166.9258	0.70641	166.4792	0.08337
0.72188	173.7987	0.56894	169.557	0.34977	167.4115	0.70906	166.9622	0.08149
0.725	174.2915	0.57004	170.057	0.3579	167.9025	0.71185	167.4506	0.07945
0.72813	174.7889	0.57137	170.5616	0.36615	168.3983	0.71478	167.9438	0.07728
0.73125	175.2902	0.57295	171.0702	0.37453	168.8984	0.71782	168.4412	0.07498
0.73438	175.7947	0.57476	171.5821	0.38304	169.4022	0.72098	168.9422	0.07257
0.7375	176.3022	0.57682	172.0971	0.3917	169.9091	0.72424	169.4464	0.07003
0.74062	176.8125	0.57914	172.6152	0.40052	170.4193	0.72759	169.9537	0.06739
0.74375	177.3257	0.58171	173.1364	0.40951	170.9327	0.731	170.4641	0.06464
0.74687	177.8418	0.58454	173.6612	0.41866	171.4495	0.73448	170.9779	0.06179
0.75	178.3609	0.58761	174.1895	0.42798	171.9698	0.73802	171.4949	0.05884

0.75313	178.8827	0.59093	174.7211	0.43749	172.4932	0.7416	172.0149	0.05581
0.75625	179.4065	0.59447	175.2555	0.44718	173.0192	0.74523	172.5373	0.05272
0.75938	179.9316	0.59821	175.7918	0.45706	173.5469	0.74889	173.0613	0.0496
0.7625	180.4568	0.60216	176.3291	0.46714	174.0752	0.75259	173.5857	0.04646
0.76563	180.9814	0.6063	176.8664	0.47745	174.6033	0.7563	174.1098	0.04332
0.76875	181.5046	0.61063	177.4032	0.48798	175.1304	0.76003	174.6329	0.04021
0.77187	182.0266	0.61517	177.9392	0.49874	175.6566	0.76375	175.1549	0.03712
0.775	182.5478	0.61991	178.4751	0.50973	176.1823	0.76746	175.6764	0.03406
0.77812	183.0694	0.62484	179.0119	0.52095	176.7085	0.77116	176.1985	0.03103
0.78125	183.5926	0.62998	179.5509	0.5324	177.2366	0.77485	176.7226	0.02803
0.78438	184.119	0.6353	180.0936	0.54406	177.7681	0.77853	177.25	0.02507
0.7875	184.6497	0.64081	180.6413	0.55592	178.3041	0.78219	177.782	0.02214
0.79063	185.1856	0.64649	181.1946	0.56797	178.8455	0.78583	178.3194	0.01926
0.79375	185.7267	0.65235	181.7539	0.5802	179.3924	0.78943	178.8623	0.01643
0.79688	186.2727	0.65836	182.3186	0.5926	179.9443	0.79296	179.4103	0.01367
0.8	186.8229	0.66453	182.888	0.60518	180.5007	0.7964	179.9626	0.01099
0.80312	187.3765	0.67084	183.4615	0.61793	181.0607	0.79971	180.5186	0.0084
0.80625	187.933	0.67725	184.0385	0.63085	181.6237	0.80288	181.0776	0.0059
0.80937	188.4921	0.68375	184.6186	0.64394	182.1896	0.80592	181.6394	0.0035
0.8125	189.0536	0.69031	185.2018	0.65719	182.7581	0.80884	182.2038	0.00121
0.81563	189.6175	0.69692	185.788	0.67059	183.3293	0.81166	182.7708	9.81E-04
0.81875	190.1837	0.70356	186.3772	0.68412	183.9031	0.81438	183.3402	0.00306
0.82188	190.7519	0.71024	186.969	0.69777	184.4792	0.81702	183.9119	0.00503
0.825	191.3219	0.71695	187.5631	0.71152	185.0573	0.81958	184.4854	0.00688
0.82813	191.8929	0.72367	188.1587	0.72535	185.6367	0.82204	185.0602	0.00863
0.83125	192.4642	0.7304	188.7549	0.73925	186.2166	0.82438	185.6355	0.01026
0.83437	193.0352	0.7371	189.3511	0.75321	186.7964	0.8266	186.2107	0.01177
0.8375	193.6053	0.74375	189.9466	0.76721	187.3754	0.82868	186.7854	0.01315
0.84062	194.1747	0.75033	190.5414	0.78127	187.9538	0.83059	187.3596	0.01439
0.84375	194.7438	0.75682	191.1358	0.79536	188.5318	0.83234	187.9336	0.01551
0.84688	195.3135	0.76321	191.7308	0.8095	189.1104	0.83392	188.5086	0.01649
0.85	195.8851	0.76949	192.3276	0.82367	189.6909	0.8353	189.0857	0.01735
0.85313	196.4601	0.77568	192.9275	0.83788	190.2745	0.83649	189.6663	0.01807
0.85625	197.0396	0.78175	193.5317	0.85211	190.8626	0.83748	190.2516	0.01862
0.85938	197.6247	0.7877	194.1411	0.86637	191.4559	0.83825	190.8426	0.01895
0.8625	198.2155	0.79349	194.7558	0.88065	192.0548	0.8388	191.4394	0.019
0.86562	198.812	0.79909	195.3756	0.89495	192.659	0.83911	192.0421	0.0187
0.86875	199.4134	0.80448	195.9996	0.90926	193.2678	0.83916	192.6498	0.01797
0.87187	200.019	0.80962	196.6271	0.92361	193.8805	0.83892	193.2619	0.01675
0.875	200.628	0.8145	197.2571	0.93798	194.4962	0.83836	193.8777	0.015
0.87813	201.2399	0.8191	197.8891	0.95238	195.1145	0.83746	194.4967	0.01268
0.88125	201.8543	0.82341	198.5227	0.96681	195.7349	0.83621	195.1185	0.0098
0.88438	202.4711	0.82741	199.1576	0.98128	196.3573	0.8346	195.7429	0.00635
0.8875	203.0901	0.83108	199.7939	0.99577	196.9815	0.83261	196.3699	0.00233
0.89063	203.7112	0.83439	200.4311	1.01028	197.6073	0.83025	196.9991	0.00228
0.89375	204.3339	0.8373	201.0689	1.02481	198.2342	0.82752	197.6303	0.00749
0.89687	204.9578	0.8398	201.7068	1.03936	198.862	0.82442	198.263	0.0133
0.9	205.5824	0.84187	202.3441	1.05391	199.4899	0.82094	198.8967	0.01972
0.90312	206.2073	0.84351	202.9804	1.06845	200.1175	0.81711	199.5309	0.02674
0.90625	206.8321	0.84475	203.6152	1.08298	200.7444	0.81292	200.1652	0.03432
0.90938	207.457	0.8456	204.2485	1.09749	201.3707	0.80841	200.7997	0.04242
0.9125	208.0823	0.84608	204.8807	1.11195	201.9966	0.80359	201.4347	0.05098
0.91563	208.7088	0.84618	205.5126	1.12636	202.6229	0.79852	202.071	0.05995
0.91875	209.3377	0.84589	206.1453	1.14069	203.2509	0.7932	202.7095	0.0693

0.92188	209.9701	0.84521	206.7801	1.15495	203.8816	0.78765	203.3517	0.07898
0.925	210.6073	0.84411	207.4183	1.16912	204.5163	0.78187	203.9986	0.08901
0.92812	211.2501	0.84259	208.0606	1.18321	205.1558	0.77585	204.6511	0.09938
0.93125	211.8989	0.84064	208.7073	1.19721	205.8005	0.76957	205.3096	0.11014
0.93437	212.5534	0.83828	209.3583	1.2111	206.4501	0.763	205.9738	0.1213
0.9375	213.213	0.83553	210.0128	1.22489	207.1041	0.75613	206.6433	0.13291
0.94063	213.8769	0.83242	210.6698	1.23854	207.7615	0.74896	207.3172	0.14497
0.94375	214.544	0.82899	211.3284	1.25205	208.4213	0.74151	207.9944	0.15747
0.94688	215.2135	0.82525	211.9877	1.2654	209.0829	0.73378	208.6742	0.17039
0.95	215.8849	0.82123	212.6473	1.27858	209.7457	0.72582	209.356	0.18367
0.95313	216.5577	0.81695	213.3068	1.29159	210.4092	0.71765	210.0395	0.19728
0.95625	217.2317	0.81246	213.966	1.30443	211.0733	0.70931	210.7243	0.21116
0.95937	217.9066	0.80778	214.6246	1.3171	211.7376	0.70082	211.4102	0.22526
0.9625	218.5822	0.80294	215.2824	1.3296	212.4019	0.6922	212.0969	0.23954
0.96562	219.2581	0.79797	215.939	1.34191	213.0658	0.68346	212.7841	0.25398
0.96875	219.934	0.79291	216.594	1.35406	213.7289	0.6746	213.4713	0.26856
0.97188	220.6098	0.78776	217.2473	1.36602	214.391	0.66562	214.1585	0.2833
0.975	221.2852	0.78255	217.8985	1.3778	215.052	0.65651	214.8455	0.29822
0.97813	221.9605	0.7773	218.5479	1.38942	215.7118	0.64729	215.5323	0.31337
0.98125	222.6359	0.77206	219.1958	1.40087	216.3709	0.63797	216.2195	0.32879
0.98438	223.3123	0.76687	219.8428	1.41218	217.03	0.62861	216.9075	0.34453
0.9875	223.9906	0.76181	220.49	1.42335	217.6898	0.61928	217.5974	0.36063
0.99062	224.6719	0.75693	221.1384	1.43438	218.3516	0.61006	218.2903	0.37714
0.99375	225.3574	0.75231	221.7893	1.44527	219.0163	0.60107	218.987	0.3941
0.99687	226.0479	0.74799	222.4434	1.456	219.6849	0.59243	219.6886	0.41153
1	226.7436	0.74401	223.1012	1.46655	220.3577	0.58423	220.3952	0.42945
1.00313	227.4445	0.74042	223.7625	1.47692	221.0345	0.57659	221.1069	0.44786
1.00625	228.1497	0.73726	224.4267	1.48707	221.7148	0.56959	221.8229	0.46677
1.00938	228.8582	0.73458	225.093	1.49701	222.3977	0.56329	222.5423	0.48613
1.0125	229.5689	0.73245	225.7603	1.50672	223.0821	0.55775	223.2641	0.50588
1.01563	230.2807	0.73095	226.4278	1.5162	223.7671	0.55301	223.9872	0.52592
1.01875	230.9928	0.73017	227.0949	1.52545	224.4518	0.5491	224.7107	0.54615
1.02188	231.7047	0.73019	227.7612	1.53446	225.1358	0.54603	225.4339	0.56641
1.025	232.4157	0.73106	228.4264	1.54323	225.8186	0.54383	226.1561	0.58657
1.02812	233.1256	0.73281	229.0906	1.55174	226.4999	0.5425	226.877	0.60651
1.03125	233.8342	0.73546	229.7537	1.55998	227.1797	0.54203	227.5962	0.62613
1.03438	234.5413	0.73899	230.4156	1.56793	227.8577	0.54241	228.3134	0.64537
1.0375	235.2468	0.74341	231.0765	1.57561	228.5339	0.54363	229.0285	0.66419
1.04063	235.9507	0.74872	231.7363	1.58299	229.2084	0.54564	229.7415	0.68255
1.04375	236.653	0.75492	232.3951	1.59008	229.8812	0.54841	230.4523	0.70042
1.04688	237.3541	0.76202	233.0531	1.59689	230.5525	0.55186	231.1611	0.71774
1.05	238.0541	0.77	233.7107	1.60344	231.2226	0.55593	231.8683	0.73448
1.05313	238.7538	0.77881	234.3685	1.60974	231.8921	0.56055	232.5744	0.75063
1.05625	239.454	0.78838	235.0275	1.61583	232.5619	0.56565	233.2801	0.76619
1.05937	240.1554	0.79864	235.6885	1.62171	233.2328	0.57117	233.9865	0.78117
1.0625	240.8592	0.80951	236.3526	1.62742	233.9058	0.57705	234.6943	0.79561
1.06563	241.5658	0.82093	237.0203	1.63298	234.5815	0.58326	235.4044	0.80955
1.06875	242.2757	0.83285	237.6921	1.6384	235.2603	0.58974	236.1171	0.82301
1.07188	242.9888	0.84526	238.3677	1.64369	235.9422	0.59645	236.8322	0.83599
1.075	243.7045	0.85812	239.0465	1.64885	236.6264	0.60331	237.5494	0.84846
1.07813	244.4218	0.87142	239.7277	1.65387	237.3121	0.61025	238.2674	0.86035
1.08125	245.1397	0.88508	240.4102	1.65875	237.9984	0.61717	238.9853	0.87158
1.08438	245.857	0.89904	241.0932	1.66349	238.6842	0.62396	239.7018	0.88204
1.0875	246.5729	0.91318	241.7759	1.66813	239.3686	0.63049	240.4159	0.89163

1.09062	247.2865	0.9274	242.4581	1.6727	240.051	0.63666	241.1268	0.90025
1.09375	247.9976	0.94163	243.1394	1.67725	240.7309	0.64237	241.834	0.90786
1.09688	248.706	0.95582	243.8199	1.68182	241.4083	0.64758	242.5371	0.91443
1.1	249.4118	0.96994	244.4996	1.68644	242.0832	0.65222	243.2363	0.91997
1.10313	250.1152	0.98399	245.1787	1.69112	242.7557	0.65628	243.9314	0.9245
1.10625	250.8164	0.99799	245.8572	1.69584	243.4259	0.65975	244.6228	0.9281
1.10938	251.5155	1.01192	246.5353	1.7006	244.0941	0.66262	245.3106	0.93081
1.1125	252.2129	1.0258	247.2131	1.70539	244.7604	0.66491	245.995	0.93273
1.11563	252.9089	1.03962	247.891	1.71021	245.4254	0.66664	246.6763	0.93392
1.11875	253.6039	1.05337	248.5692	1.71505	246.0892	0.66781	247.3551	0.93444
1.12187	254.2983	1.06707	249.2484	1.71995	246.7526	0.66841	248.0317	0.93432
1.125	254.9926	1.08072	249.9292	1.72491	247.4161	0.66841	248.7067	0.93353
1.12813	255.6876	1.09431	250.6124	1.72995	248.0804	0.66774	249.3808	0.93203
1.13125	256.3838	1.10783	251.2986	1.73507	248.7463	0.6663	250.0546	0.92972
1.13438	257.0817	1.12121	251.9885	1.74028	249.4143	0.66402	250.7286	0.92652
1.1375	257.7815	1.13438	252.6824	1.74557	250.0849	0.66081	251.403	0.92231
1.14063	258.483	1.14725	253.3803	1.75095	250.7579	0.6566	252.0777	0.91701
1.14375	259.1855	1.1597	254.0816	1.75644	251.433	0.65135	252.7522	0.91051
1.14688	259.8881	1.17166	254.7857	1.76205	252.1092	0.64506	253.4256	0.90276
1.15	260.5896	1.18306	255.4914	1.7678	252.7857	0.63772	254.0969	0.8937
1.15312	261.2891	1.19388	256.1978	1.77374	253.4612	0.62934	254.7651	0.88328
1.15625	261.9857	1.20408	256.9039	1.77987	254.135	0.61995	255.4293	0.87147
1.15938	262.6788	1.21367	257.6092	1.7862	254.8064	0.60957	256.0887	0.85823
1.1625	263.3683	1.22263	258.3132	1.79274	255.4751	0.59823	256.7431	0.84355
1.16563	264.054	1.23094	259.0158	1.79946	256.1409	0.586	257.3924	0.82741
1.16875	264.7362	1.23859	259.7171	1.80635	256.804	0.57297	258.0368	0.80985
1.17188	265.4152	1.24555	260.4172	1.81339	257.4649	0.55929	258.6766	0.79093
1.175	266.0916	1.25182	261.1163	1.82056	258.1237	0.54512	259.3125	0.77073
1.17813	266.7657	1.25741	261.8146	1.82784	258.781	0.53068	259.9451	0.74936
1.18125	267.438	1.26234	262.5121	1.83523	259.4372	0.51617	260.575	0.72693
1.18437	268.109	1.26666	263.2089	1.84272	260.0925	0.50181	261.2028	0.70352
1.1875	268.7791	1.2704	263.9052	1.85031	260.7472	0.48779	261.8289	0.6792
1.19063	269.4487	1.27357	264.6011	1.85801	261.4017	0.47431	262.4538	0.65403
1.19375	270.1182	1.27615	265.297	1.86582	262.0564	0.46158	263.0781	0.62804
1.19688	270.7883	1.27808	265.9934	1.87375	262.7117	0.4498	263.7023	0.60129
1.2	271.4592	1.27932	266.6907	1.88177	263.3681	0.43915	264.3268	0.57381
1.20313	272.1315	1.27984	267.3894	1.88988	264.0262	0.42981	264.9524	0.5456
1.20625	272.8054	1.27966	268.0897	1.89803	264.6863	0.42195	265.5792	0.51667
1.20938	273.4806	1.27883	268.7915	1.90619	265.3482	0.41575	266.2073	0.48702
1.2125	274.1567	1.27741	269.4944	1.91432	266.0116	0.4114	266.8361	0.45662
1.21563	274.8329	1.2755	270.1975	1.9224	266.6756	0.40914	267.4649	0.42547
1.21875	275.508	1.27314	270.9	1.93041	267.3395	0.4092	268.0927	0.3936
1.22188	276.181	1.27038	271.6007	1.93835	268.002	0.41182	268.7183	0.36106
1.225	276.8509	1.26721	272.2989	1.94623	268.6623	0.4172	269.3409	0.32796
1.22813	277.5169	1.26363	272.9939	1.95403	269.3197	0.42548	269.9596	0.29442
1.23125	278.1786	1.25965	273.6853	1.96174	269.9737	0.43669	270.574	0.26055
1.23438	278.8359	1.2553	274.3729	1.96932	270.6241	0.45078	271.1842	0.2265
1.2375	279.4891	1.25067	275.0569	1.97674	271.2712	0.46759	271.7904	0.19239
1.24063	280.1384	1.24584	275.7373	1.98392	271.9153	0.48692	272.3932	0.15834
1.24375	280.7845	1.2409	276.4143	1.99081	272.5568	0.50848	272.9931	0.12445
1.24688	281.4277	1.2359	277.0882	1.99735	273.1961	0.53198	273.5908	0.09082
1.25	282.0684	1.23084	277.7591	2.0035	273.8337	0.55708	274.1868	0.05752
1.25313	282.7066	1.22571	278.4273	2.00925	274.4697	0.58341	274.7814	0.02463
1.25625	283.3425	1.22046	279.0929	2.0146	275.1044	0.61064	275.3751	0.00782

1.25938	283.9762	1.21505	279.7562	2.01958	275.738	0.63844	275.968	0.03981
1.2625	284.6079	1.20946	280.4176	2.02418	276.3708	0.66654	276.5605	0.0714
1.26563	285.2378	1.2037	281.0775	2.02843	277.0029	0.69475	277.1528	0.10268
1.26875	285.8659	1.19774	281.7362	2.0323	277.6347	0.72292	277.7454	0.13375
1.27188	286.4926	1.19155	282.394	2.03581	278.2665	0.75094	278.3385	0.16472
1.275	287.118	1.18506	283.0512	2.03894	278.8983	0.77872	278.9323	0.19562
1.27813	287.742	1.17819	283.7076	2.0417	279.53	0.80615	279.5267	0.22642
1.28125	288.3643	1.17087	284.3628	2.0441	280.1614	0.83308	280.1215	0.25705
1.28438	288.9842	1.1631	285.0161	2.04615	280.7914	0.85937	280.7161	0.28735
1.2875	289.601	1.1549	285.6665	2.04785	281.4193	0.88485	281.3094	0.31718
1.29063	290.2137	1.14642	286.3129	2.0492	282.0438	0.9094	281.9007	0.34641
1.29375	290.8217	1.13782	286.9542	2.05018	282.6641	0.93293	282.4891	0.37495
1.29688	291.4242	1.1293	287.5898	2.05075	283.2792	0.95542	283.0737	0.40273
1.3	292.0211	1.12101	288.219	2.05089	283.889	0.9769	283.6544	0.4297
1.30313	292.6123	1.11306	288.8418	2.05056	284.4933	0.99742	284.2311	0.45584
1.30625	293.1979	1.10551	289.458	2.04972	285.0923	1.01702	284.8039	0.48112
1.30938	293.7785	1.09834	290.068	2.04836	285.6864	1.03571	285.3732	0.50549
1.3125	294.3543	1.09155	290.6719	2.04649	286.2762	1.0535	285.9397	0.52892
1.31563	294.9257	1.08512	291.2702	2.04412	286.862	1.07035	286.5036	0.55137
1.31875	295.4931	1.07904	291.8629	2.04129	287.4442	1.08627	287.0655	0.57283
1.32188	296.0564	1.07332	292.4504	2.03804	288.0231	1.10127	287.6254	0.59332
1.325	296.6158	1.068	293.0328	2.03441	288.5986	1.11542	288.1834	0.6129
1.32813	297.1712	1.06305	293.61	2.03046	289.1709	1.12882	288.7394	0.63165
1.33125	297.7226	1.05846	294.1824	2.02626	289.7399	1.14158	289.2932	0.64967
1.33438	298.2697	1.05418	294.7502	2.02186	290.3058	1.1538	289.8446	0.66705
1.3375	298.8125	1.05017	295.3138	2.01731	290.8686	1.16557	290.3936	0.68386
1.34063	299.351	1.0464	295.8735	2.01262	291.4287	1.17695	290.9401	0.70014
1.34375	299.8849	1.04287	296.4295	2.0078	291.9859	1.188	291.484	0.71593
1.34688	300.4141	1.03962	296.9817	2.00284	292.5403	1.19876	292.0252	0.73121
1.35	300.9381	1.03672	297.5299	1.99774	293.0915	1.20929	292.5631	0.74597
1.35313	301.456	1.03422	298.0732	1.99247	293.6387	1.21967	293.0972	0.76015
1.35625	301.9671	1.03218	298.6107	1.98703	294.1813	1.22994	293.6264	0.77369
1.35938	302.4704	1.0306	299.1413	1.98145	294.7182	1.24011	294.1498	0.78652
1.3625	302.9652	1.02945	299.664	1.97572	295.2485	1.25017	294.6666	0.79861
1.36563	303.4508	1.0287	300.178	1.96983	295.7717	1.26007	295.1761	0.80995
1.36875	303.927	1.0283	300.6828	1.96375	296.2874	1.26976	295.678	0.82058
1.37188	304.3938	1.02823	301.1783	1.95744	296.7955	1.27916	296.1724	0.83054
1.375	304.8516	1.02855	301.6645	1.95081	297.2962	1.28818	296.6598	0.83987
1.37813	305.301	1.02934	302.1419	1.94383	297.7902	1.29672	297.1408	0.84856
1.38125	305.7426	1.03077	302.6106	1.93645	298.2781	1.30467	297.6163	0.85659
1.38438	306.1773	1.03302	303.0712	1.92867	298.7604	1.31194	298.0867	0.8639
1.3875	306.6054	1.03626	303.524	1.92056	299.2376	1.31842	298.5528	0.87043
1.39063	307.0272	1.04067	303.9691	1.91217	299.71	1.32402	299.0146	0.87616
1.39375	307.4429	1.04636	304.4068	1.90357	300.1776	1.32872	299.4723	0.88108
1.39688	307.8524	1.05345	304.837	1.89481	300.6404	1.33253	299.9258	0.88527
1.4	308.2556	1.06202	305.2599	1.8859	301.0984	1.33552	300.3749	0.88879
