# Supplementary material for: Detection of DNA oligonucleotides with base mutations by terahertz spectroscopy and microstructures
Source: PLoS One. 2018 Jan 24;13(1):e0191515. doi: 10.1371/journal.pone.0191515 (PMC5783420; doi:10.1371/journal.pone.0191515)
Supplement: S4 File — (PDF) [file pone.0191515.s004.pdf]

Plotting Data of Fig. 5

Frequency (THz)	Transmission									
	Ter-5A	SD	Ter-5C	SD	Ter-5T	SD	Ter-5G	SD	SRRs	SD
1.22089	0.1545	0.003323	0.19828	0.001961	0.16603	0.002461	0.18715	0.002086	0.21848	0.002204
1.22209	0.15339	0.003275	0.1972	0.00195	0.16471	0.002429	0.1861	0.002062	0.21745	0.002204
1.22329	0.15232	0.00323	0.1961	0.00194	0.16342	0.0024	0.18506	0.00204	0.21639	0.002206
1.22449	0.15129	0.003188	0.19497	0.001933	0.16216	0.002373	0.184	0.00202	0.21529	0.00221
1.22569	0.1503	0.003151	0.19383	0.001926	0.16091	0.002349	0.18294	0.002002	0.21416	0.002215
1.22689	0.14934	0.003116	0.19265	0.001921	0.1597	0.002327	0.18188	0.001987	0.213	0.00222
1.22809	0.14841	0.003085	0.19146	0.001916	0.1585	0.002308	0.1808	0.001973	0.2118	0.002224
1.22929	0.14752	0.003057	0.19025	0.001912	0.15734	0.002291	0.17973	0.001962	0.21058	0.002228
1.23049	0.14666	0.003033	0.18901	0.001908	0.15619	0.002277	0.17865	0.001952	0.20933	0.002232
1.23169	0.14583	0.003011	0.18776	0.001905	0.15506	0.002264	0.17756	0.001944	0.20805	0.002236
1.23289	0.14503	0.002991	0.18649	0.001902	0.15396	0.002253	0.17647	0.001938	0.20674	0.002239
1.23409	0.14425	0.002974	0.1852	0.001899	0.15288	0.002244	0.17538	0.001933	0.20541	0.002241
1.23529	0.14351	0.002958	0.1839	0.001896	0.15181	0.002236	0.17428	0.001929	0.20405	0.002243
1.23649	0.14279	0.002945	0.18258	0.001893	0.15077	0.002229	0.17318	0.001926	0.20266	0.002243
1.2377	0.14209	0.002932	0.18124	0.00189	0.14975	0.002223	0.17207	0.001923	0.20126	0.002243
1.2389	0.14142	0.002922	0.17989	0.001887	0.14874	0.002219	0.17097	0.001922	0.19983	0.002242
1.2401	0.14076	0.002912	0.17853	0.001883	0.14775	0.002215	0.16986	0.001921	0.19838	0.00224
1.2413	0.14013	0.002903	0.17715	0.001879	0.14678	0.002211	0.16874	0.001921	0.19691	0.002237
1.2425	0.13952	0.002894	0.17576	0.001874	0.14583	0.002208	0.16763	0.00192	0.19542	0.002233
1.2437	0.13892	0.002887	0.17437	0.001869	0.14489	0.002205	0.16651	0.00192	0.19391	0.002228
1.2449	0.13835	0.002879	0.17296	0.001863	0.14397	0.002202	0.16539	0.00192	0.19238	0.002222
1.2461	0.13778	0.00287	0.17154	0.001856	0.14306	0.002198	0.16427	0.001919	0.19083	0.002214
1.2473	0.13723	0.002862	0.17012	0.001849	0.14217	0.002195	0.16314	0.001918	0.18927	0.002205
1.2485	0.1367	0.002853	0.16868	0.00184	0.14129	0.002191	0.16202	0.001916	0.1877	0.002195
1.2497	0.13617	0.002843	0.16724	0.001831	0.14042	0.002185	0.16089	0.001913	0.18611	0.002184
1.2509	0.13566	0.002831	0.1658	0.00182	0.13957	0.002179	0.15977	0.00191	0.18451	0.00217
1.2521	0.13515	0.002818	0.16435	0.001808	0.13873	0.002172	0.15864	0.001905	0.18289	0.002156
1.2533	0.13462	0.002804	0.16289	0.001795	0.1379	0.002164	0.15752	0.0019	0.18126	0.00214
1.2545	0.13407	0.002788	0.16143	0.001781	0.13708	0.002153	0.15639	0.001892	0.17963	0.002123
1.2557	0.13352	0.00277	0.15997	0.001766	0.13627	0.002141	0.15526	0.001883	0.17798	0.002104
1.2569	0.13296	0.002749	0.15851	0.001749	0.13547	0.002128	0.15414	0.001873	0.17632	0.002084

1.2581	0.13239	0.002726	0.15705	0.001731	0.13468	0.002113	0.15301	0.001861	0.17466	0.002061
1.2593	0.13181	0.0027	0.15558	0.00171	0.1339	0.002095	0.15189	0.001846	0.17299	0.002037
1.2605	0.13122	0.00267	0.15412	0.001688	0.13313	0.002074	0.15077	0.001829	0.17132	0.002011
1.2617	0.13061	0.002638	0.15266	0.001665	0.13229	0.002052	0.14964	0.001811	0.16964	0.001984
1.26291	0.12999	0.002601	0.1512	0.00164	0.13144	0.002026	0.14853	0.001789	0.16795	0.001954
1.26411	0.12936	0.002561	0.14974	0.001613	0.13059	0.001997	0.14741	0.001765	0.16626	0.001922
1.26531	0.12872	0.002518	0.14829	0.001584	0.12972	0.001966	0.14629	0.001738	0.16457	0.001888
1.26651	0.12807	0.002469	0.14684	0.001552	0.12885	0.001931	0.14521	0.001708	0.16288	0.001852
1.26771	0.1274	0.002417	0.1454	0.001519	0.12797	0.001893	0.14412	0.001675	0.16119	0.001814
1.26891	0.12672	0.002359	0.14397	0.001483	0.12709	0.001851	0.14302	0.001639	0.1595	0.001774
1.27011	0.12603	0.002297	0.14254	0.001444	0.1262	0.001805	0.14192	0.001599	0.15781	0.001731
1.27131	0.12533	0.002229	0.14112	0.001403	0.1253	0.001756	0.14081	0.001556	0.15612	0.001686
1.27251	0.12462	0.002156	0.13971	0.001359	0.1244	0.001702	0.1397	0.001509	0.15444	0.001637
1.27371	0.12391	0.002077	0.13831	0.001312	0.1235	0.001644	0.13858	0.001458	0.15276	0.001587
1.27491	0.12319	0.001992	0.13692	0.001262	0.1226	0.001581	0.13746	0.001403	0.15109	0.001534
1.27611	0.12247	0.001901	0.13555	0.001209	0.1217	0.001514	0.13634	0.001344	0.14942	0.001478
1.27731	0.12174	0.001804	0.13438	0.001154	0.1208	0.001443	0.13521	0.001281	0.14776	0.001419
1.27851	0.12102	0.001703	0.13322	0.001096	0.1199	0.001368	0.13409	0.001216	0.14637	0.001359
1.27971	0.12029	0.001596	0.13205	0.001036	0.11901	0.00129	0.13296	0.001147	0.14498	0.001297
1.28091	0.11957	0.001487	0.13089	0.000975	0.11812	0.00121	0.13184	0.001076	0.14358	0.001233
1.28211	0.11886	0.001375	0.12972	0.000912	0.11724	0.001128	0.13072	0.001004	0.14217	0.001169
1.28331	0.11815	0.001259	0.12854	0.000849	0.11638	0.001044	0.1296	0.00093	0.14076	0.001104
1.28451	0.11745	0.001143	0.12737	0.000786	0.11552	0.000959	0.12849	0.000856	0.13933	0.001038
1.28571	0.11677	0.001026	0.12621	0.000723	0.11468	0.000875	0.12739	0.000782	0.1379	0.000973
1.28691	0.1161	0.000909	0.12504	0.000661	0.11386	0.000791	0.12629	0.000709	0.13645	0.000908
1.28812	0.11544	0.000791	0.12388	0.0006	0.11305	0.000708	0.1252	0.000636	0.135	0.000844
1.28932	0.11481	0.000676	0.12273	0.000541	0.11227	0.000628	0.12412	0.000566	0.13355	0.000782
1.29052	0.1142	0.000562	0.12159	0.000485	0.11151	0.000552	0.12306	0.0005	0.13209	0.000722
1.29172	0.11361	0.00045	0.12047	0.000432	0.11077	0.000479	0.12201	0.000437	0.13063	0.000663
1.29292	0.11304	0.000342	0.11937	0.000383	0.11006	0.000414	0.12097	0.00038	0.12918	0.000608
1.29412	0.11251	0.000236	0.11828	0.000339	0.10938	0.000356	0.11996	0.000329	0.12773	0.000554
1.29532	0.112	0.000137	0.11722	0.000302	0.10873	0.000309	0.11896	0.000288	0.1263	0.000505
1.29652	0.11153	4.09E-05	0.11618	0.000272	0.10811	0.000277	0.11798	0.000258	0.12488	0.00046
1.29772	0.11109	4.87E-05	0.11517	0.000251	0.10753	0.000263	0.11703	0.000242	0.12348	0.000419
1.29892	0.11069	0.00013	0.11419	0.000239	0.10698	0.000264	0.1161	0.000239	0.1221	0.000383

1.30012	0.11033	0.000207	0.11324	0.000236	0.10648	0.000278	0.1152	0.000248	0.12074	0.000352
1.30132	0.11001	0.000275	0.11233	0.00024	0.10601	0.0003	0.11433	0.000264	0.11941	0.000328
1.30252	0.10974	0.000336	0.11144	0.00025	0.10559	0.000325	0.11349	0.000283	0.1181	0.00031
1.30372	0.1095	0.000392	0.11059	0.000266	0.10521	0.000353	0.11267	0.000306	0.11682	0.000298
1.30492	0.10932	0.000443	0.10978	0.000284	0.10488	0.00038	0.11189	0.000328	0.11557	0.000294
1.30612	0.10918	0.00049	0.109	0.000306	0.10459	0.000408	0.11115	0.000351	0.11434	0.000298
1.30732	0.10909	0.000533	0.10825	0.00033	0.10436	0.000433	0.11044	0.000373	0.11315	0.000307
1.30852	0.10905	0.000576	0.10755	0.000355	0.10417	0.000459	0.10976	0.000395	0.11199	0.000322
1.30972	0.10906	0.000617	0.10688	0.000381	0.10403	0.000484	0.10913	0.000417	0.11087	0.000342
1.31092	0.10912	0.000658	0.10625	0.000409	0.10394	0.00051	0.10853	0.000438	0.10978	0.000366
1.31212	0.10924	0.000699	0.10566	0.000436	0.1039	0.000535	0.10797	0.000458	0.10873	0.000392
1.31333	0.10941	0.000742	0.1051	0.000464	0.1039	0.000562	0.10746	0.000479	0.10771	0.000421
1.31453	0.10964	0.000788	0.10459	0.000492	0.10396	0.00059	0.10699	0.0005	0.10673	0.000451
1.31573	0.10992	0.000835	0.10412	0.00052	0.10407	0.000619	0.10656	0.000522	0.10579	0.000483
1.31693	0.11025	0.000884	0.10368	0.000548	0.10422	0.000649	0.10617	0.000544	0.10488	0.000516
1.31813	0.11064	0.000933	0.10329	0.000576	0.10443	0.00068	0.10582	0.000567	0.10402	0.00055
1.31933	0.11107	0.000985	0.10294	0.000605	0.10468	0.000713	0.10553	0.000591	0.1032	0.000584
1.32053	0.11156	0.001038	0.10263	0.000634	0.10497	0.000746	0.10527	0.000616	0.10242	0.00062
1.32173	0.1121	0.001091	0.10236	0.000662	0.10531	0.000781	0.10506	0.000642	0.10169	0.000655
1.32293	0.11268	0.001146	0.10213	0.000691	0.1057	0.000816	0.1049	0.000669	0.10099	0.000691
1.32413	0.1133	0.001201	0.10194	0.00072	0.10613	0.000853	0.10478	0.000698	0.10034	0.000727
1.32533	0.11397	0.001256	0.10179	0.00075	0.10659	0.00089	0.10471	0.000727	0.09974	0.000763
1.32653	0.11468	0.001312	0.10168	0.00078	0.1071	0.000929	0.10469	0.000758	0.09918	0.0008
1.32773	0.11542	0.001368	0.10162	0.00081	0.10764	0.000968	0.1047	0.00079	0.09866	0.000837
1.32893	0.1162	0.001424	0.10159	0.00084	0.10822	0.001007	0.10477	0.000824	0.0982	0.000874
1.33013	0.11701	0.001479	0.1016	0.00087	0.10884	0.001046	0.10487	0.000857	0.09777	0.000911
1.33133	0.11785	0.001535	0.10165	0.000901	0.10948	0.001086	0.10502	0.000892	0.09739	0.000948
1.33253	0.11871	0.001591	0.10174	0.000932	0.11016	0.001127	0.10521	0.000928	0.09706	0.000985
1.33373	0.11959	0.001645	0.10187	0.000962	0.11087	0.001167	0.10545	0.000964	0.09678	0.001021
1.33493	0.12049	0.001699	0.10204	0.000993	0.1116	0.001206	0.10572	0.001	0.09654	0.001057
1.33613	0.12141	0.001751	0.10225	0.001023	0.11236	0.001246	0.10603	0.001037	0.09635	0.001093
1.33733	0.12234	0.001803	0.10249	0.001053	0.11315	0.001285	0.10638	0.001074	0.0962	0.001128
1.33854	0.12327	0.001853	0.10277	0.001083	0.11396	0.001323	0.10676	0.00111	0.0961	0.001163
1.33974	0.12421	0.001903	0.10308	0.001113	0.11479	0.001361	0.10718	0.001147	0.09604	0.001197
1.34094	0.12516	0.00195	0.10343	0.001142	0.11564	0.001398	0.10763	0.001183	0.09603	0.001231

1.34214	0.12611	0.001995	0.10382	0.00117	0.11652	0.001433	0.10811	0.001219	0.09607	0.001263
1.34334	0.12705	0.002039	0.10424	0.001197	0.11741	0.001468	0.10861	0.001254	0.09615	0.001294
1.34454	0.128	0.00208	0.1047	0.001224	0.11832	0.001502	0.10913	0.001288	0.09627	0.001325
1.34574	0.12894	0.002121	0.10519	0.00125	0.11925	0.001534	0.10968	0.001321	0.09644	0.001354
1.34694	0.12988	0.002157	0.10571	0.001274	0.1202	0.001564	0.11025	0.001353	0.09665	0.001382
1.34814	0.13081	0.002192	0.10627	0.001297	0.12116	0.001593	0.11084	0.001383	0.09691	0.001408
1.34934	0.13173	0.002223	0.10686	0.001319	0.12214	0.001619	0.11144	0.001412	0.0972	0.001433
1.35054	0.13265	0.002253	0.10749	0.001339	0.12313	0.001644	0.11205	0.001439	0.09754	0.001457
1.35174	0.13356	0.002278	0.10815	0.001358	0.12414	0.001666	0.11267	0.001464	0.09792	0.001478
1.35294	0.13447	0.0023	0.10884	0.001374	0.12516	0.001686	0.11331	0.001487	0.09833	0.001498
1.35414	0.13536	0.00232	0.10957	0.001389	0.1262	0.001704	0.11395	0.001508	0.09879	0.001516
1.35534	0.13625	0.002336	0.11033	0.001402	0.12724	0.001719	0.11461	0.001526	0.09928	0.001532
1.35654	0.13713	0.002348	0.11112	0.001412	0.1283	0.001731	0.11526	0.001542	0.09981	0.001545
1.35774	0.138	0.002356	0.11194	0.00142	0.12937	0.00174	0.11593	0.001555	0.10037	0.001556
1.35894	0.13887	0.00236	0.1128	0.001425	0.13045	0.001746	0.1166	0.001564	0.10097	0.001565
1.36014	0.13973	0.00236	0.11369	0.001428	0.13154	0.001749	0.11728	0.001571	0.1016	0.001571
1.36134	0.14059	0.002356	0.11461	0.001428	0.13264	0.001748	0.11796	0.001575	0.10226	0.001574
1.36255	0.14144	0.002348	0.11556	0.001425	0.13375	0.001745	0.11864	0.001575	0.10296	0.001575
1.36375	0.14229	0.002334	0.11654	0.001419	0.13487	0.001737	0.11934	0.001571	0.10368	0.001573
1.36495	0.14314	0.002317	0.11756	0.001411	0.136	0.001727	0.12004	0.001565	0.10443	0.001569
1.36615	0.14399	0.002296	0.1186	0.0014	0.13713	0.001713	0.12075	0.001555	0.10521	0.001562
1.36735	0.14484	0.002272	0.11968	0.001387	0.13827	0.001697	0.12147	0.001544	0.10602	0.001553
1.36855	0.1457	0.002245	0.12079	0.001372	0.13941	0.001679	0.1222	0.001529	0.10685	0.001543
1.36975	0.14655	0.002216	0.12192	0.001356	0.14056	0.001659	0.12294	0.001514	0.10771	0.001531
1.37095	0.14741	0.002186	0.12308	0.001339	0.14172	0.001638	0.1237	0.001497	0.1086	0.001519
1.37215	0.14828	0.002154	0.12427	0.001321	0.14288	0.001616	0.12447	0.001479	0.10951	0.001506
1.37335	0.14916	0.002121	0.12548	0.001303	0.14404	0.001594	0.12526	0.00146	0.11045	0.001492
1.37455	0.15004	0.002089	0.12672	0.001285	0.14521	0.001571	0.12607	0.001442	0.1113	0.001479
1.37575	0.15093	0.002057	0.12782	0.001268	0.14638	0.001549	0.1269	0.001424	0.11218	0.001466
1.37695	0.15183	0.002025	0.12895	0.001251	0.14756	0.001528	0.12774	0.001406	0.11308	0.001454
1.37815	0.15273	0.001996	0.13009	0.001236	0.14875	0.001507	0.12861	0.00139	0.11401	0.001443
1.37935	0.15365	0.001967	0.13124	0.001222	0.14994	0.001489	0.1295	0.001376	0.11497	0.001433
1.38055	0.15459	0.001941	0.13242	0.00121	0.15113	0.001471	0.13041	0.001362	0.11595	0.001425
1.38175	0.15553	0.001918	0.13362	0.0012	0.15233	0.001457	0.13134	0.001352	0.11695	0.001418
1.38295	0.15648	0.001897	0.13483	0.001192	0.15354	0.001444	0.13229	0.001344	0.11798	0.001414

1.38415	0.15745	0.001882	0.13606	0.001188	0.15475	0.001436	0.13327	0.001339	0.11903	0.001412
1.38535	0.15844	0.00187	0.13731	0.001187	0.15597	0.00143	0.13426	0.001338	0.1201	0.001413
1.38655	0.15943	0.001863	0.13858	0.00119	0.15719	0.001429	0.13519	0.001341	0.1212	0.001418
1.38776	0.16044	0.00186	0.13986	0.001196	0.15842	0.001431	0.13614	0.001348	0.12233	0.001425
1.38896	0.16147	0.001864	0.14117	0.001207	0.15966	0.001439	0.13711	0.00136	0.12347	0.001437
1.39016	0.16251	0.001873	0.14249	0.001222	0.1609	0.001451	0.13809	0.001376	0.12465	0.001451
1.39136	0.16356	0.001886	0.14382	0.00124	0.16214	0.001465	0.13909	0.001396	0.12584	0.001469
1.39256	0.16463	0.001902	0.14518	0.001261	0.16339	0.001483	0.1401	0.001419	0.12706	0.001489
1.39376	0.16572	0.00192	0.14655	0.001284	0.16464	0.001503	0.14113	0.001444	0.1283	0.001511
1.39496	0.16682	0.001942	0.14794	0.001309	0.16589	0.001526	0.14218	0.001471	0.12957	0.001534
1.39616	0.16793	0.001964	0.14934	0.001335	0.16715	0.001549	0.14325	0.0015	0.13086	0.001558
1.39736	0.16906	0.001988	0.15076	0.001362	0.16842	0.001573	0.14433	0.001529	0.13217	0.001583
1.39856	0.1702	0.002011	0.1522	0.001389	0.16968	0.001597	0.14543	0.001559	0.13351	0.001608
1.39976	0.17145	0.002035	0.15365	0.001417	0.17096	0.001622	0.14655	0.001589	0.13487	0.001633
1.40096	0.17271	0.002056	0.15512	0.001443	0.17223	0.001645	0.14769	0.001618	0.13625	0.001658
1.40216	0.17397	0.002076	0.15661	0.001468	0.17351	0.001667	0.14884	0.001646	0.13766	0.001681
1.40336	0.17523	0.002093	0.15811	0.00149	0.17479	0.001686	0.15002	0.001672	0.13908	0.001702
1.40456	0.17649	0.002107	0.15963	0.001511	0.17607	0.001704	0.15121	0.001695	0.14053	0.001721
1.40576	0.17775	0.002117	0.16116	0.001529	0.17735	0.001718	0.15242	0.001716	0.14201	0.001737
1.40696	0.17902	0.002123	0.16271	0.001543	0.17864	0.001728	0.15365	0.001733	0.14351	0.001751
1.40816	0.18028	0.002124	0.16427	0.001553	0.17992	0.001735	0.1549	0.001747	0.14502	0.001761
1.40936	0.18153	0.002117	0.16584	0.001559	0.18121	0.001736	0.15617	0.001755	0.14657	0.001767
1.41056	0.18278	0.002106	0.16744	0.001561	0.1825	0.001733	0.15746	0.001759	0.14813	0.001768
1.41176	0.18403	0.002087	0.16904	0.001556	0.18379	0.001724	0.15877	0.001758	0.14972	0.001765
1.41297	0.18527	0.002059	0.17066	0.001546	0.18508	0.001708	0.1601	0.00175	0.15132	0.001756
1.41417	0.1865	0.002024	0.1723	0.001531	0.18637	0.001686	0.16145	0.001736	0.15295	0.001743
1.41537	0.18772	0.001981	0.17395	0.00151	0.18766	0.001658	0.16281	0.001716	0.15461	0.001724
1.41657	0.18892	0.00193	0.17561	0.001484	0.18895	0.001624	0.1642	0.001691	0.15628	0.001701
1.41777	0.19012	0.001874	0.17729	0.001453	0.19024	0.001584	0.16561	0.00166	0.15798	0.001673
1.41897	0.1913	0.00181	0.17898	0.001419	0.19153	0.00154	0.16704	0.001625	0.1597	0.001641
1.42017	0.19247	0.001742	0.18068	0.001382	0.19281	0.001492	0.16849	0.001586	0.16144	0.001607
