# Supplementary material for: Detection of DNA oligonucleotides with base mutations by terahertz spectroscopy and microstructures
Source: PLoS One. 2018 Jan 24;13(1):e0191515. doi: 10.1371/journal.pone.0191515 (PMC5783420; doi:10.1371/journal.pone.0191515)
Supplement: S5 File — (PDF) [file pone.0191515.s005.pdf]

Plotting Data of Fig. 6a

	Numer of H-Bonds			
Time(ns)	Ter-5A	Ter-5C	Ter-5T	Ter-5G
0.05	1.89E+17	1.78E+17	1.77E+17	1.92E+17
0.1	1.79E+17	1.86E+17	1.85E+17	1.88E+17
0.15	1.74E+17	1.93E+17	1.73E+17	1.83E+17
0.2	1.88E+17	1.79E+17	1.68E+17	1.73E+17
0.25	1.73E+17	1.87E+17	1.85E+17	1.96E+17
0.3	1.75E+17	1.90E+17	1.73E+17	1.89E+17
0.35	1.78E+17	2.01E+17	1.71E+17	1.93E+17
0.4	1.85E+17	1.97E+17	1.73E+17	1.89E+17
0.45	1.82E+17	1.90E+17	1.67E+17	1.97E+17
0.5	1.83E+17	1.79E+17	1.75E+17	1.97E+17
0.55	1.86E+17	1.89E+17	1.75E+17	1.92E+17
0.6	1.83E+17	1.94E+17	1.81E+17	1.74E+17
0.65	1.82E+17	1.82E+17	1.76E+17	1.68E+17
0.7	1.87E+17	1.77E+17	1.83E+17	1.83E+17
0.75	1.98E+17	1.82E+17	1.73E+17	1.88E+17
0.8	1.86E+17	1.88E+17	1.77E+17	1.95E+17
0.85	1.85E+17	1.70E+17	1.74E+17	1.88E+17
0.9	1.90E+17	1.95E+17	1.74E+17	1.89E+17
0.95	1.84E+17	1.93E+17	1.75E+17	1.82E+17
1	1.95E+17	1.96E+17	1.79E+17	1.86E+17
1.05	1.78E+17	1.96E+17	1.62E+17	1.77E+17
1.1	1.87E+17	1.92E+17	1.72E+17	1.88E+17
1.15	1.83E+17	1.77E+17	1.79E+17	1.91E+17
1.2	1.84E+17	1.90E+17	1.68E+17	2.00E+17
1.25	1.86E+17	1.91E+17	1.69E+17	1.89E+17
1.3	1.91E+17	1.78E+17	1.83E+17	1.88E+17
1.35	1.90E+17	1.97E+17	1.78E+17	1.78E+17
1.4	1.92E+17	1.79E+17	1.69E+17	1.88E+17
1.45	1.92E+17	1.99E+17	1.56E+17	1.74E+17
1.5	1.88E+17	1.78E+17	1.77E+17	1.81E+17
1.55	1.83E+17	1.85E+17	1.71E+17	1.91E+17
1.6	1.75E+17	1.79E+17	1.77E+17	1.82E+17
1.65	1.83E+17	2.01E+17	1.75E+17	1.87E+17
1.7	1.86E+17	1.79E+17	1.78E+17	1.88E+17
1.75	1.88E+17	1.83E+17	1.81E+17	1.98E+17
1.8	1.87E+17	1.95E+17	1.69E+17	1.78E+17
1.85	1.75E+17	1.94E+17	1.79E+17	1.73E+17
1.9	1.73E+17	1.94E+17	1.69E+17	1.80E+17
1.95	1.96E+17	1.96E+17	1.76E+17	1.89E+17
2	1.81E+17	1.97E+17	1.82E+17	2.00E+17
2.05	1.83E+17	1.99E+17	1.69E+17	1.96E+17
2.1	1.75E+17	2.13E+17	1.75E+17	1.83E+17
2.15	1.97E+17	1.85E+17	1.77E+17	2.02E+17
2.2	1.85E+17	1.86E+17	1.74E+17	1.88E+17
2.25	1.87E+17	1.81E+17	1.73E+17	1.83E+17
2.3	1.83E+17	2.06E+17	1.80E+17	1.90E+17
2.35	1.84E+17	1.89E+17	1.69E+17	1.84E+17
2.4	1.84E+17	1.90E+17	1.80E+17	1.85E+17
2.45	1.77E+17	1.82E+17	1.73E+17	2.00E+17

2.5	1.84E+17	1.75E+17	1.68E+17	1.92E+17
2.55	1.77E+17	1.84E+17	1.69E+17	1.86E+17
2.6	2.01E+17	1.83E+17	1.64E+17	1.83E+17
2.65	1.79E+17	1.93E+17	1.82E+17	1.94E+17
2.7	1.78E+17	1.87E+17	1.79E+17	1.91E+17
2.75	1.95E+17	1.85E+17	1.81E+17	1.96E+17
2.8	1.91E+17	1.83E+17	1.68E+17	2.00E+17
2.85	1.87E+17	1.88E+17	1.88E+17	1.97E+17
2.9	1.82E+17	1.93E+17	1.74E+17	1.86E+17
2.95	1.69E+17	1.84E+17	1.84E+17	1.97E+17
3	1.79E+17	1.83E+17	1.67E+17	1.90E+17
3.05	1.84E+17	1.88E+17	1.66E+17	1.95E+17
3.1	1.79E+17	1.96E+17	1.78E+17	2.12E+17
3.15	1.89E+17	1.82E+17	1.78E+17	1.85E+17
3.2	1.83E+17	1.81E+17	1.76E+17	1.77E+17
3.25	1.98E+17	1.79E+17	1.67E+17	1.85E+17
3.3	1.83E+17	1.78E+17	1.69E+17	1.85E+17
3.35	1.76E+17	1.95E+17	1.80E+17	1.81E+17
3.4	1.83E+17	1.92E+17	1.71E+17	1.90E+17
3.45	1.96E+17	1.92E+17	1.89E+17	1.90E+17
3.5	1.79E+17	1.89E+17	1.71E+17	1.96E+17
3.55	1.69E+17	1.99E+17	1.80E+17	1.94E+17
3.6	1.90E+17	1.89E+17	1.78E+17	1.90E+17
3.65	1.82E+17	1.78E+17	1.71E+17	1.88E+17
3.7	1.76E+17	1.95E+17	1.81E+17	2.03E+17
3.75	1.75E+17	1.83E+17	1.72E+17	1.81E+17
3.8	1.80E+17	1.87E+17	1.79E+17	1.96E+17
3.85	1.84E+17	1.70E+17	1.78E+17	1.96E+17
3.9	1.77E+17	1.82E+17	1.74E+17	1.89E+17
3.95	1.80E+17	1.86E+17	1.79E+17	1.85E+17
4	1.83E+17	1.85E+17	1.73E+17	1.83E+17
4.05	1.87E+17	1.89E+17	1.85E+17	1.79E+17
4.1	1.84E+17	1.81E+17	1.77E+17	1.88E+17
4.15	1.80E+17	1.95E+17	1.86E+17	1.83E+17
4.2	1.81E+17	1.93E+17	1.84E+17	1.88E+17
4.25	1.65E+17	1.84E+17	1.84E+17	1.90E+17
4.3	1.83E+17	1.96E+17	1.79E+17	1.77E+17
4.35	1.64E+17	1.93E+17	1.68E+17	1.82E+17
4.4	1.84E+17	1.72E+17	1.79E+17	1.93E+17
4.45	1.71E+17	1.81E+17	1.70E+17	1.77E+17
4.5	1.99E+17	1.90E+17	1.76E+17	1.97E+17
4.55	1.98E+17	1.84E+17	1.68E+17	1.95E+17
4.6	1.74E+17	1.93E+17	1.77E+17	1.96E+17
4.65	2.00E+17	1.85E+17	1.83E+17	1.98E+17
4.7	2.04E+17	1.96E+17	1.76E+17	1.88E+17
4.75	1.78E+17	1.90E+17	1.80E+17	1.81E+17
4.8	1.65E+17	2.04E+17	1.85E+17	1.89E+17
4.85	1.85E+17	1.83E+17	1.64E+17	1.77E+17
4.9	1.88E+17	1.81E+17	1.71E+17	1.85E+17
4.95	2.02E+17	1.88E+17	1.72E+17	1.88E+17
5	1.71E+17	1.92E+17	1.65E+17	1.87E+17
5.05	1.82E+17	1.89E+17	1.70E+17	1.91E+17
5.1	1.80E+17	1.84E+17	1.58E+17	1.91E+17
5.15	1.86E+17	1.93E+17	1.82E+17	1.90E+17

5.2	1.86E+17	1.94E+17	1.74E+17	1.74E+17
5.25	1.80E+17	1.78E+17	1.76E+17	1.99E+17
5.3	1.88E+17	1.82E+17	1.74E+17	1.76E+17
5.35	1.86E+17	1.92E+17	1.71E+17	1.91E+17
5.4	1.85E+17	1.77E+17	1.76E+17	1.85E+17
5.45	1.75E+17	1.91E+17	1.77E+17	1.91E+17
5.5	1.78E+17	2.00E+17	1.80E+17	1.73E+17
5.55	1.84E+17	1.97E+17	1.79E+17	1.81E+17
5.6	1.69E+17	1.90E+17	1.76E+17	1.74E+17
5.65	1.82E+17	1.90E+17	1.85E+17	1.76E+17
5.7	1.82E+17	1.82E+17	1.66E+17	1.88E+17
5.75	1.86E+17	1.95E+17	1.85E+17	1.79E+17
5.8	1.74E+17	1.68E+17	1.77E+17	1.73E+17
5.85	1.75E+17	1.97E+17	1.85E+17	1.83E+17
5.9	1.75E+17	1.80E+17	1.73E+17	1.83E+17
5.95	1.95E+17	1.58E+17	1.80E+17	1.92E+17
6	1.71E+17	1.89E+17	1.69E+17	1.73E+17
6.05	1.78E+17	1.77E+17	1.75E+17	1.92E+17
6.1	1.80E+17	1.88E+17	1.75E+17	1.74E+17
6.15	1.78E+17	1.79E+17	1.83E+17	1.77E+17
6.2	1.80E+17	1.88E+17	1.71E+17	1.83E+17
6.25	1.75E+17	1.86E+17	1.72E+17	1.86E+17
6.3	1.57E+17	1.72E+17	1.72E+17	1.85E+17
6.35	1.78E+17	1.92E+17	1.85E+17	1.72E+17
6.4	1.83E+17	1.86E+17	1.83E+17	1.77E+17
6.45	1.80E+17	1.79E+17	1.84E+17	1.84E+17
6.5	1.67E+17	1.70E+17	1.79E+17	1.81E+17
6.55	1.76E+17	1.81E+17	1.73E+17	1.81E+17
6.6	1.78E+17	1.88E+17	1.76E+17	1.80E+17
6.65	1.79E+17	1.78E+17	1.76E+17	1.93E+17
6.7	1.78E+17	1.80E+17	1.81E+17	1.90E+17
6.75	1.79E+17	1.82E+17	1.79E+17	1.87E+17
6.8	1.73E+17	1.83E+17	1.69E+17	1.78E+17
6.85	1.88E+17	1.92E+17	1.81E+17	1.78E+17
6.9	1.88E+17	1.93E+17	1.81E+17	1.79E+17
6.95	1.77E+17	1.92E+17	1.79E+17	1.83E+17
7	1.71E+17	1.96E+17	1.77E+17	1.94E+17
7.05	1.84E+17	1.79E+17	1.80E+17	1.68E+17
7.1	1.68E+17	1.80E+17	1.75E+17	1.63E+17
7.15	1.77E+17	1.77E+17	1.73E+17	1.79E+17
7.2	1.75E+17	1.72E+17	1.66E+17	1.74E+17
7.25	1.86E+17	1.81E+17	1.84E+17	1.87E+17
7.3	1.92E+17	1.76E+17	1.87E+17	1.84E+17
7.35	1.82E+17	1.67E+17	1.71E+17	1.70E+17
7.4	1.89E+17	1.96E+17	1.67E+17	1.68E+17
7.45	1.88E+17	1.78E+17	1.89E+17	1.85E+17
7.5	1.85E+17	1.81E+17	1.85E+17	1.89E+17
7.55	1.78E+17	1.92E+17	1.84E+17	1.85E+17
7.6	1.92E+17	1.82E+17	1.76E+17	1.91E+17
7.65	1.83E+17	1.90E+17	1.87E+17	1.83E+17
7.7	1.70E+17	1.76E+17	1.80E+17	1.90E+17
7.75	1.80E+17	1.92E+17	1.73E+17	1.85E+17
7.8	1.78E+17	1.83E+17	1.89E+17	1.76E+17
7.85	1.84E+17	1.79E+17	1.71E+17	1.82E+17

7.9	1.74E+17	1.97E+17	1.79E+17	1.89E+17
7.95	1.89E+17	1.79E+17	1.79E+17	1.85E+17
8	1.72E+17	1.77E+17	1.77E+17	1.81E+17
8.05	1.84E+17	1.88E+17	1.68E+17	1.74E+17
8.1	1.79E+17	1.91E+17	1.59E+17	1.88E+17
8.15	1.87E+17	1.84E+17	1.75E+17	1.90E+17
8.2	1.74E+17	1.82E+17	1.80E+17	1.74E+17
8.25	1.81E+17	1.93E+17	1.68E+17	1.86E+17
8.3	1.88E+17	1.85E+17	1.71E+17	1.89E+17
8.35	1.80E+17	1.90E+17	1.80E+17	1.83E+17
8.4	1.81E+17	1.90E+17	1.79E+17	1.90E+17
8.45	1.75E+17	1.79E+17	1.84E+17	1.87E+17
8.5	1.91E+17	2.00E+17	1.77E+17	1.84E+17
8.55	1.88E+17	1.81E+17	1.72E+17	1.88E+17
8.6	1.84E+17	1.83E+17	1.81E+17	1.88E+17
8.65	1.97E+17	1.96E+17	1.86E+17	1.82E+17
8.7	1.97E+17	1.78E+17	1.84E+17	1.95E+17
8.75	1.91E+17	1.93E+17	1.72E+17	1.78E+17
8.8	1.87E+17	1.74E+17	1.67E+17	1.79E+17
8.85	1.69E+17	1.84E+17	1.91E+17	1.90E+17
8.9	1.86E+17	1.89E+17	1.73E+17	1.85E+17
8.95	1.86E+17	1.82E+17	1.77E+17	1.86E+17
9	1.86E+17	1.82E+17	1.64E+17	1.78E+17
9.05	1.76E+17	1.92E+17	1.74E+17	1.87E+17
9.1	1.93E+17	1.80E+17	1.82E+17	1.92E+17
9.15	1.87E+17	1.74E+17	1.61E+17	1.81E+17
9.2	1.94E+17	1.81E+17	1.71E+17	1.79E+17
9.25	1.79E+17	1.87E+17	1.91E+17	1.96E+17
9.3	1.75E+17	1.74E+17	2.00E+17	1.93E+17
9.35	1.76E+17	1.89E+17	1.88E+17	1.93E+17
9.4	1.94E+17	1.87E+17	1.67E+17	1.78E+17
9.45	1.94E+17	1.80E+17	1.88E+17	1.86E+17
9.5	1.90E+17	1.63E+17	1.84E+17	1.93E+17
9.55	1.87E+17	1.84E+17	1.83E+17	1.82E+17
9.6	1.97E+17	1.82E+17	1.93E+17	1.96E+17
9.65	1.94E+17	1.75E+17	1.80E+17	1.85E+17
9.7	2.05E+17	1.87E+17	1.66E+17	1.99E+17
9.75	1.87E+17	1.96E+17	1.73E+17	1.91E+17
9.8	1.81E+17	1.84E+17	1.77E+17	1.92E+17
9.85	1.96E+17	1.85E+17	1.80E+17	1.84E+17
9.9	1.89E+17	1.85E+17	1.73E+17	1.85E+17
9.95	1.72E+17	1.96E+17	1.85E+17	1.86E+17
10	1.80E+17	1.95E+17	1.83E+17	1.86E+17
