# Supplementary material for: Detection of DNA oligonucleotides with base mutations by terahertz spectroscopy and microstructures
Source: PLoS One. 2018 Jan 24;13(1):e0191515. doi: 10.1371/journal.pone.0191515 (PMC5783420; doi:10.1371/journal.pone.0191515)
Supplement: S6 File — (PDF) [file pone.0191515.s006.pdf]

Plotting Data of Fig. 6b

| | Average | SD |
|---|---|---|
| TA-T | 1.76E+17 | 1.86E+15 |
| TA-A | 1.83E+17 | 2.88E+15 |
| TA-G | 1.86E+17 | 3.19E+15 |
| TA-C | 1.86E+17 | 2.62E+15 |
